# Synthesis of α,ω-bis-Mercaptoacyl Poly(alkyl oxide)s and Development of Thioether Cross-Linked Liposome Scaffolds for Sustained Release of Drugs

**DOI:** 10.3390/molecules29061312

**Published:** 2024-03-15

**Authors:** Spyridon Mourtas, Georgios Kourmoulakis, Stavros Kremezis, Pavlos Klepetsanis, Sophia G. Antimisiaris

**Affiliations:** 1 Department of Chemistry, University of Patras, 26510 Rio Patras, Greece; 2 Laboratory of Pharmaceutical Technology, Department of Pharmacy, School of Health Sciences, University of Patras, 26510 Rio Patras, Greece; geokourmoulakis93@gmail.com (G.K.); stauros_chemeng@yahoo.com (S.K.); klepe@upatras.gr (P.K.); 3 Institute of Chemical Engineering Sciences, Foundation for Research and Technology Hellas (FORTH/ICES), 26504 Rio Patras, Greece

**Keywords:** *S*-4-methoxytityl mercapto acids, α,ω-homofunctional mercaptoacyl poly(alkyl oxide)s, thia–Michael addition, thioether cross-linked liposomes

## Abstract

With the aim to develop novel scaffolds for the sustained release of drugs, we initially developed an easy approach for the synthesis of α,ω-homobifunctional mercaptoacyl poly(alkyl oxide)s. This was based on the esterification of the terminal hydroxyl groups of poly(alkyl oxide)s with suitably *S*-4-methoxytrityl (Mmt)-protected mercapto acids, followed by the removal of the acid labile *S*-Mmt group. This method allowed for the efficient synthesis of the title compounds in high yield and purity, which were further used in the development of a thioether cross-linked liposome scaffold, by thia–Michael reaction of the terminal thiol groups with pre-formed nano-sized liposomes bearing maleimide groups on their surface. The reaction process was followed by ^1^H-NMR, using a Carr–Purcell–Meiboom–Gill (CPMG) relaxation dispersion NMR experiment (^1^H-NMR CPMG), which allowed for real-time monitoring and optimization of the reaction process. The thioether cross-linked liposomal scaffold that was synthesized was proven to preserve the nano-sized characteristics of the initial liposomes and allowed for the sustained release of calcein (which was used as a hydrophilic dye and a hydrophilic drug model), providing evidence for the efficient synthesis of a novel drug release scaffold consisting of nanoliposome building blocks.

## 1. Introduction

**Figure 1:**
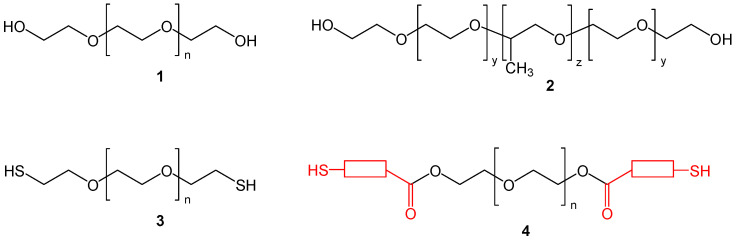
General structure of poly(ethylene glycol) **1**, pluronic **2**, dithiol poly(ethylene glycol) **3,** and homobifunctional mercaptoacyl poly(ethylene glycol) **4**.

Poly(alkyl oxide)s (PAOs) are biomacromolecules with the general formula HO-(CH_2_-CHR-O)x-H. Poly(ethylene glycol) (PEG) **1** (Figure 1), where the R group of PAOs is H, also known as poly(ethylene oxide) (PEO) or poly(oxyethylene) (POE), depending on its molecular weight, is a synthetic linear polyether with wide range of biomedical applications, due to its excellent water solubility, biocompatibility, low toxicity, low immunogenicity and antigenicity. In addition, PEG is non-biodegradable, yet it is readily excretable after administration into living organisms, exhibiting excellent pharmacokinetic and biodistribution behavior. All these properties make PEG particularly attractive and an ideal linker to graft organic molecules and biomolecules. In addition, PEG-conjugated drugs have been approved by the U.S. Food and Drug Administration (FDA) for safe use in humans [1,2,3,4].

Other types of PAOs, such as pluronics **2** (Figure 1), also represent an important class of biomacromolecules. They are composed of poly(ethylene glycol)–block–poly(propylene glycol)–block–poly(ethylene glycol), also abbreviated as “triblock PEO-PPO-PEO copolymers”, where PPO represents poly(propylene oxide) chains. The PEO block is considered hydrophilic and water-soluble, while the PPO block is hydrophobic and water-insoluble. The combination of these hydrophilic/lipophilic chains introduces special properties to these polymers, enabling their self-assembly, and their use as drug carriers [5,6,7].

Both PEG and pluronic have been widely functionalized on their hydroxyl groups with a variety of groups or molecules, increasing their applicability. In fact, hetero and homobifunctional PEG derivatives are very useful, acting as cross-linking agents or spacers between two chemical entities [8]. In particular, the homobifunctional dithiol PEG derivatives of type **3** and **4** (Figure 1), which represent synthetic polyethers containing two end-group thiol functionalities, are of high interest, as they could be used in simple and rapid crosslinking reactions with drugs, peptides or proteins, based on the properties of a thiol group to act as a nucleophile, mainly by the thiol–maleimide method, where the thiol group is used as a Michael donor [9,10,11], or in thiol–ene click chemistry [12,13]. In particular, the homofunctional dithiol PEGs of type **4**, where thiol-containing building blocks have been introduced into the hydroxyl end groups of the PEG molecules, have been used as alternatives to dithiol PEGs of type **3** [14,15,16,17]. Such derivatives (**4**) can be synthesized more easily and have the advantage of structural diversity.

Despite the high interest, the commercial availability of homobifunctional dithiol PEGs derived from **1** (and the more complicated **2**) is extremely low and the cost is very high. In fact, by searching the commercial availability of dithiol PEGs, we found that it is limited to the replacement of the hydroxyl end groups by the thiol group (type **3** molecules), while the commercial availability of homobifunctional dithiols of type **4** are limited to 3-mercaptopropionate derivatives, with pentaerythritol tetrakis(3-mercaptopropionate) being the most representative. Regarding the methods that have been proposed for the synthesis of type **4** molecules, they are not attractive, and they have several drawbacks, i.e., sophisticated equipment and harsh reaction conditions, such as long reaction times, high temperatures, high excess of mercapto acids and catalysts, as well as the need of high amounts of dithiothreitol (DTT) in parallel with the use of nitrogen flow during the reaction [18,19,20,21,22,23,24,25,26,27,28,29]. Therefore, the introduction of new methods for the synthesis of type **4** molecules, overcoming the above limitations, is a challenge. Thus, in the first part of this work, we considered the synthesis of α,ω-bis-mercaptoacyl poly(alkyl oxide)s of type **4**, by a simple and efficient method. In the next part of this work, the synthesized compounds were used in the development of a drug-release scaffold consisted of thioether cross-linked nanoliposomes. Liposomes are mainly consisted of phospholipids and cholesterol; thus, among the various nanoparticle types, they are usually preferred as drug carriers because they are structurally versatile, highly biocompatible, and non-toxic [30,31]. Liposome pegylation, which refers to the coating of the vesicle surface with polyethylene glycol molecules, can efficiently reduce the rapid clearance of liposomes by macrophages and prolong their circulation time in the blood [32,33]. Thus, the synthesis of cross-linked nanoliposomes, by the reaction of type **4** molecules with nanoliposomes bearing maleimide groups on their surface, was considered to be a method to synthesize novel and stable liposome scaffolds for sustained release of drugs. To this end, we efficiently synthesized a liposomal scaffold, which was proven to maintain the nano-sized characteristics of the initial liposome form. We also proved that the synthesized scaffold enables sustained release of a hydrophilic dye (calcein), which is also used as a model of hydrophilic drugs, thus proving its potential use as a novel drug release scaffold consisting of nanoliposome building blocks.

## 2. Results and Discussion

### 2.1. Rationale

In order to synthesize innovating cross-linked nano-sized liposomes, we considered the use of homofunctional poly(alkyl oxide)s as a cross-linking agent. Searching the literature, we found the preparation of liposome-cross-linked hybrids by the cross-linking reaction of end-functionalized 4-arm PEG-thiols and maleimide functionalized liposomes, through a thia–Michael type addition. Using this method, liposome-cross-linked hybrid hydrogels were formed as the result of extensive cross-linking between the end-functionalized 4-arm PEG-dithiols and maleimide-functionalized liposomes [34].

Inspired by this work, we designed a novel cross-linked liposomal scaffold for the sustained release of drugs, where the initial nano-sized characteristics of liposomes would be preserved after the cross-linking reaction. To this end, we considered the replacement of 4-arm PEG-dithiols with α,ω-bis-mercapto poly(alkyl oxide)s as a structural modification that would reduce the extensive cross-linking and allow for the development of nano-sized scaffolds (instead of liposome-cross-linked hybrid hydrogels).

In this approach, we considered the use of high-molecular-weight end-type PEG-dithiols, such as α,ω-bis-mercaptoacyl poly(alkyl oxide)s, as molecular tools of particular interest. However, the synthetic approaches that have been developed so far for the synthesis of α,ω-bis-mercapto poly(alkyl oxide)s are not attractive and need to be replaced by simple and more efficient methods, avoiding high temperatures, sophisticated equipment, oxidation problems, and by-products formation. Thus, a novel method for the synthesis of α,ω-bis-mercaptoacyl poly(alkyl oxide)s was initially developed, which was further used in the synthesis of the proposed nano-sized scaffold.

### 2.2. Synthesis of Functionalized α,ω-bis-Mercaptoacyl Poly(alkyl oxide)s

#### 2.2.1. Selection and Synthesis of *S*-Mmt Mercapto Acids

**Figure 2:**
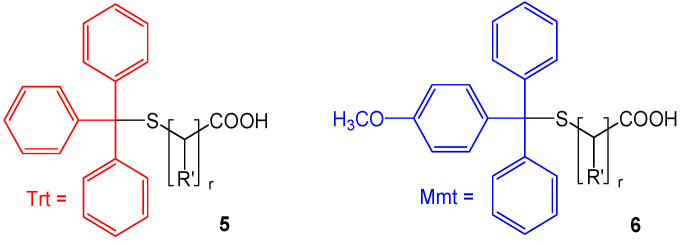
General structure of *S*-Trt mercapto acids **5** and *S*-Mmt mercapto acids **6**. R = 1, R′ = CH_3_; r = 2–5, R′ = H.

For the synthesis of α,ω-bis-mercaptoacyl poly(alkyl oxide)s, we considered the esterification of the hydroxyl end groups of poly(alkyl oxide)s with suitably *S*-protected mercapto acids. Among the several protecting groups of thiols, the trityl-type protecting groups, such as trityl (Trt) and 4-methoxytrityl (Mmt), are very attractive. In fact, both groups are well known for their acid lability; however, the *S*-Trt-group requires treatment with concentrated trifluoroacetic acid (TFA) and is reversible, while the use of the *S*-Mmt group has been proposed as the very acid-labile alternative of *S*-Trt-protecting group, allowing simple and effective removal of the *S*-Mmt group even with 1–3% TFA in dichloromethane (DCM) and triethyl silane (TES) [35]. Thus, we previously reported methods for the synthesis of *S*-Mmt mercapto acids **6** as the very acid-labile alternative of *S*-Trt mercapto acids **5** [36] (Figure 2).

**Scheme 1:**
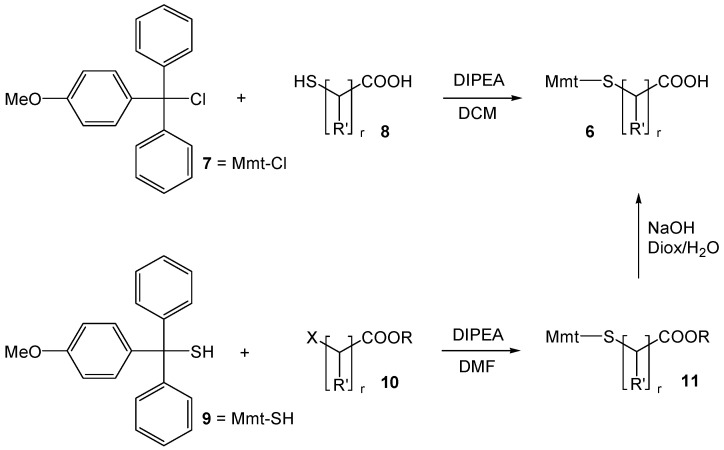
Methods for the synthesis of **6**; r = 1, R′ = CH_3_; r = 2–5, R′ = H.

In the first method, 4-methoxytrityl chloride (Mmt-Cl) **7** is reacted with commercially available mercapto acids **8**, in the presence of *N,N*′-diisopropylethylamine (DIPEA), while in the second method, 4-methoxytrityl thiol (Mmt-SH) **9** is reacted with halo acids or esters **10** to initially form the *S*-Mmt-mercapto esters **11**, which are further hydrolyzed to afford the *S*-Mmt-mercapto acids **6** (Scheme 1).

For the synthesis of α,ω-bis-mercaptoacyl poly(alkyl oxide)s, we considered the synthesis of two *S*-Mmt-mercaptoacids: (a) *S*-Mmt-2-mercaptopropionic acid (**6a**; R′ = CH_3_; r = 1) and (b) *S*-Mmt-3-mercaptopropionic acid (**6b**; R′ = H; r = 2). Regarding the method of choice for their synthesis, considering that both mercapto acids are commercially available, the first method was chosen. For the synthesis, 2-mercaptopropionic acid (thiolactic acid) (**8a**; R′ = CH_3_; r = 1) and 3-mercaptopropionic acid (**8b**; R′ = H; r = 2) were reacted with Mmt-Cl **7** in the presence of DIPEA, and **6a**/**b** were isolated in high yield and purity, as confirmed by NMR and HPLC analysis (Figures S1–S5). These compounds (**6a**/**b**) were further used in the synthesis of α,ω-bis-2-mercaptopropionyl polyethylene glycols (abbreviated as PEG-dithiols hereafter) and α,ω-bis-3-mercaptopropionyl poly(ethylene glycol)–block–poly(propylene glycol)–block–poly(ethylene glycol) copolymers (abbreviated as pluronic-dithiols hereafter).

#### 2.2.2. Synthesis of di-*S*-Mmt-PEG-Dithiols and PEG-Dithiols

**Scheme 2:**
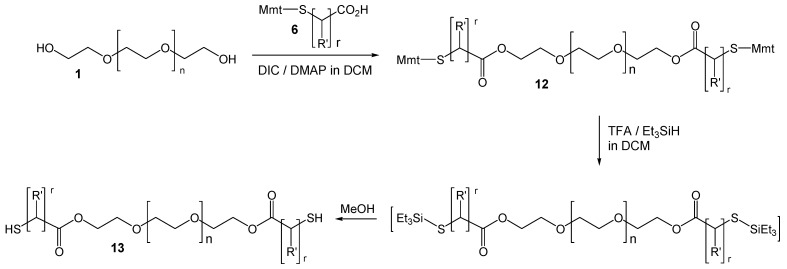
Method for the synthesis of homobifunctional di-*S*-Mmt-PEG-dithiols (**12**) and PEG-dithiols (**13**).

The proposed synthetic approach is shown in Scheme 2. In brief, we propose the reaction of PEG **1** with *S*-Mmt mercapto acids **6** by the Steglich esterification method. This requires the use of *N,N*′-diisoproplylcarbodiimide (DΙC) and 4-(dimethylamino)pyridine (DMAP) as the carboxylic acid activating system (Scheme 2) [37,38] to initially afford the *S*-Mmt-mercaptoacylated poly(ethylene glycol)s **12** (abbreviated as di-*S*-Mmt-PEG-dithiols hereafter). In this approach, the use of *S*-Mmt-protecting group during the esterification reaction has a double role, which is to (a) prevent oxidation of the thiol groups of mercapto acids to the corresponding disulfides and (b) prevent the formation of thioesters and/or thiolactones (which would be formed due to inter-molecular, and/or intra-molecular nucleophilic attack of the free thiol groups to the activated carboxylic acid groups) and/or polymerization processes (due to the formation of active thioester/thiolactone intermediates, along with the presence of free thiol groups) [39,40,41]. In addition, there are two obvious advantages after the esterification reaction as follows: (a) prevent disulfide formation by initially obtaining the *S*-Mmt-protected dithiols, ultimately increasing potential storage times, a common problem associated with thiol-containing compounds and (b) allow an easy deprotection of the *S*-Mmt group. In fact, treatment of **12** with 1–3% TFA and TES in DCM enables fast and irreversible cleavage of the *S*-Mmt group. The use of TES is known to allow irreversible removal of the *S*-Mmt group by donating a hydride to the resulting Mmt cation and driving the equilibrium towards cleavage [42]. It is reasonable that the presence of TES in the cleavage mixture, or during concentration of the cleavage mixture, may also form the corresponding sililated product, due to the formation of Si-S bonds. However, although thiosilanes are thermally stable, the Si-S bond is readily cleaved by many protic solvents, particularly those containing the O-H group [43]. Thus, in order to ensure complete cleavage of Si-S bonds of silylthioethers, further treatment with a suitable solvent, such as methanol (MeOH), is required, to enable silane alcoholysis and afford the desired PEG-dithiol **13**.

**Figure 3:**
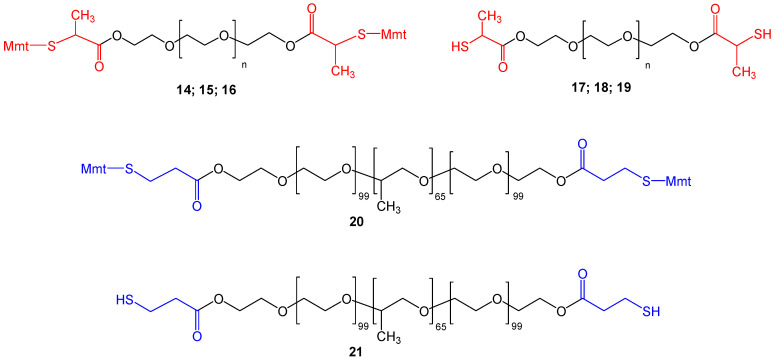
di-*S*-Mmt-PEG-dithiols **14** (PEG 10.000), **15** (PEG 4.000), **16** (PEG 1.000), and the corresponding PEG-dithiols (**17**–**19**); di-*S*-Mmt-pluronic-dithiol (**20**) and pluronic-dithiol (**21**) that were synthesized.

By using this method, we were able to obtain the *S*-Mmt-PEG-dithiols **14** (PEG 10.000), **15** (PEG 4.000), and **16** (PEG 1.000), which, upon treatment with TFA in DCM/TES (95:5) and further treatment with MeOH, afforded the corresponding deprotected PEG-dithiols **17** (PEG 10.000), **18** (PEG 4.000), and **19** (PEG 1.000) (Figure 3). All products (**14–19**) were obtained in high yield and purity, as confirmed by ^1^H and ^13^C-NMR analysis (Figures S6–S15). In addition, the ^1^H-NMR analysis clearly indicated the quantitative esterification of both hydroxyl end groups of PEG with the applied *S*-Mmt mercapto acids and quantitative removal of *S*-Mmt-protecting groups. ESI-MS analysis of **17**, **18,** and **19** was also indicative of the synthesized products (Figures S16–S18).

#### 2.2.3. Synthesis of di-*S*-Mmt-Pluronic-Dithiols and Pluronic-Dithiols

Besides the linear poly(ethylene glycol) (PEG) polymer, our method was also applied in the esterification of the more complicated thermosensitive polyoxyethylene–polyoxypropylene triblock copolymer (PEO-PPO-PEO). For this, we used pluronic F127, which is composed of Poly(ethylene glycol)_100_–Poly(propylene glycol)_65_–Poly(ethylene glycol)_100_. This was acylated with *S*-Mmt-3-mercaptopropionic acid (**6**; R′ = H; r = 2), which resulted in the synthesis of the di-*S*-Mmt-di-3-mercaptopropionyl pluronic **20** in high yield and purity. Cleavage of the *S*-Mmt group with TFA in DCM/TES (95:5) and further treatment with MeOH (as previously explained), resulted in α,ω-di-3-mercaptopropionyl pluronic **21** in high yield and purity. ^1^H and ^13^C-NMR analysis was indicative of the synthesized products and proved the complete esterification of both hydroxyl end groups (Figures S19–S22). ESI-MS spectrum analysis was also performed and indicates the successful synthesis of **21** (Figure S23).

### 2.3. Thia–Michael Reaction with Pre-Formed Liposomes Maleimides

#### 2.3.1. General Method

**Scheme 3:**
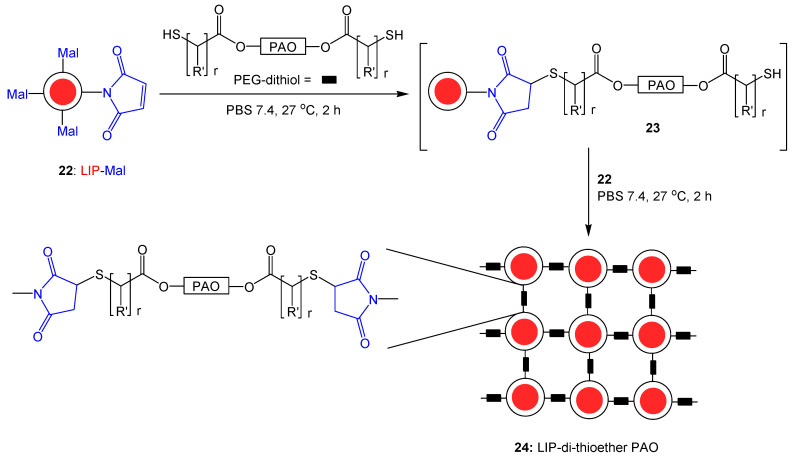
Thia–Michael reaction of α,ω-bis-mercaptoacyl poly(alkyl oxide) (PAO-dithiol) with nano-sized liposomes bearing maleimide groups on their surface, to afford a nano-sized cross-linked thioether-type liposomal scaffold. In our case, di-2-mercaptopropionyl PEG10.000 **17** and nano-sized SUV-type liposomes were used.

The general idea for the synthesis of nano-sized cross-linked liposomes is shown in Scheme 3. According to this approach, α,ω-bis-mercaptoacyl poly(alkyl oxides) (PAO-dithiols) react with pre-formed nano-sized liposomes **22** bearing maleimide groups on their surface, under thia–Michael addition conditions. The presence of a liposome dispersion in the reaction mixture enables not only the linkage of one end group of PAO-dithiol with one maleimide group, as shown in the non-isolated intermediate **23**, but also allows for further reaction of the second available thiol group (of the initially formed **23**) with a second available maleimide group. Obviously, the presence of maleimide groups on every liposomal particle enables a multi-directed thia–Michael reaction, which ensures the formation of a cross-linked liposomal scaffold consisting of thioether groups and poly(alkyl oxide) linkages between liposomes (**24**).

#### 2.3.2. Monitoring of the Reaction Progress by 1D CPMG ^1^H-NMR; Optimization

For the synthesis of the thioether liposome scaffold **24**, we chose the high-molecular-weight di-2-mercaptopropionyl PEG10.000 **17** (abbreviated as PEG-dithiol hereafter), to be reacted with nano-sized SUV-type (Small Unilamellar Vesicles) liposomes, which consisted of phosphatidylcholine (PC), cholesterol (Chol), and 1,2-distearoyl-sn-glycero-3-phosphoethanolamine-*N*-[maleimide(polyethylene glycol)-2000] (ammonium salt) (DSPE-PEG2000-Mal) in a molar ratio of PC/Chol/DSPE-PEG2000-Mal 2:1:0.03 mol/mol (abbreviated as PC-Lip-Mal hereafter), and the reaction progress of PEG-dithiol with the surface-available maleimide groups was monitored, in real-time conditions, using a Carr–Purcell–Meiboom–Gill (CPMG) relaxation dispersion NMR experiment (1D CPMG ^1^H-NMR). This pulse sequence is known to eliminate the broad proton resonances of high molecular weight macromolecules (such as proteins, lipids, polysaccharides, and other macromolecules), facilitating the observation of the signals of low molecular weight molecules [44,45,46]. By applying this method directly to pre-formed PC-Lip-Mal liposomes, we were able to identify a single sharp peak at 6.81 ppm, which corresponds to the protons of the maleimide group that is present on the surface of liposomes (Figure S24). This was a clear indication that a CPMG relaxation dispersion NMR experiment could be useful for monitoring the surface-available maleimide protons, and, therefore, the reaction progress of PEG-dithiol with surface-available maleimide groups. Next, we investigated the chemical stability of PC-Lip-Mal liposomes over hydrolysis of maleimide groups when incubated in D_2_O at 27 °C and a liposome concentration of 20 mg/mL (the conditions for the reaction of PEG-dithiol with liposome maleimides). The results showed that maleimide groups are not hydrolyzed for at least 3 h, indicating that liposome maleimides remain at their activated form and may react with PEG-dithiol for at least this time period. The ^1^H-NMR data for this set of experiments can be viewed in Figure S25.

Finally, PEG-dithiol and PC-Lip-Mal were reacted in aqueous media (D_2_O). For this reaction, the lipid concentration was also adjusted at 20 mg/mL, and liposomes were incubated at 27 °C (the conditions of our initial experiments) with a 2.5 molar excess of PEG-dithiol (with respect to maleimide groups). The reaction progress was monitored by ^1^H-NMR using the CPMG experiment. The results showed that the peak at 6.93 ppm, which corresponds to maleimide protons, was decreased in time, and finally disappeared within 2 h, indicating that all available maleimide groups have been reacted with the PEG-dithiol in this time period. The time-dependent ^1^H-NMR analysis of the reaction mixture (real-time monitoring of the reaction progress) can be viewed in Figure S26.

### 2.4. Effect of Thioether Cross-Linking on Liposome Size

In order to investigate the effect of the molar excess of PEG-dithiol and the effect of the concentration of the reaction mixture (i.e., liposome concentration), on the size of the liposomal system that is formed, we tested (a) different excesses of PEG-dithiol with respect to maleimide groups (2.5, 5.0, and 10.0 times molar excess), and (b) two liposome concentrations (of 20 and 40 mg/mL). In addition, two liposome compositions were prepared: PC-Lips-Mal SUV liposomes consisted of PC/Chol/DSPE-PEG2000-Mal (2:1:0.03) mol/mol and control liposomes (without maleimide groups) consisted of PC/Chol/DSPE-PEG2000-OMe (2:1:0.03) mol/mol. The physicochemical characteristics of the synthesized systems are provided in Table 1 (for 20 mg/mL) and Table 2 (for 40 mg/mL).

As it is seen, increasing the excess of PEG-dithiol and lipid concentration, there is a significant effect on the hydrodynamic diameter (size) of PC-Lips-Mal, possibly because more extensive cross-linking between the PEG-dithiol and maleimide groups takes place. In brief, at 20 mg/mL lipid concentration, the size of the control liposomes (without maleimide) did not change during their reaction with PEG-dithiol (105.1 vs. 101.8 nm), while the size of the corresponding PC-Lip-Mal increased about 30% (from 101.8 to 128.90 nm) for 2.5 molar excess of PEG-dithiol. A 50% increase (from 101.8 to 153.10 nm) was seen for the highest tested PEG-dithiol excess (×10). Similar behavior was seen for the PC-Lip-Mal of 40 mg/mL, where the size of the control liposomes (without maleimide groups) remained unchanged during the incubation of liposomes with PEG-dithiol (89.38 vs. 90.93 nm), while the PC-Lips-Mal showed about 40% increase for the 2.5 molar excess (from 90.93 to 138.30 nm) and about 50% increase for the highest tested excess (×10) (from 90.93 to 148 nm). Based on these findings, it can be safely said that the increase in size of PC-Lips-Mal, upon incubation with PEG-dithiol, is attributed to the reaction of PEG-dithiol with the surface-available maleimide groups of PC-Lip-Mal, which results in size increment.

The polydispersity index (PDI) is another important parameter for the homogeneity of a sample. Although the incubation of PEG-dithiol with control liposomes (without maleimide groups) had no effect on their size distribution, the reaction of PC-Lip-Mal with 2.5 molar excess (at 20 mg/mL) resulted in a PDI value of 0.199, slightly higher than the initial PDI value, indicative of a homogeneous sample. The corresponding value for the sample of 40 mg/mL was slightly higher (0.244), indicating a less homogenous sample. Higher excesses of PEG-dithiol (5.0 and 10.0 times relative to the maleimide group) resulted in a considerable increase in the polydispersity index (>0.25). The ζ-potential of all liposome structures (before and after the addition of PEG-dithiol) ranged between −2.20 and −2.65 mV.

### 2.5. Scanning Electron Microscope (SEM) Images

**Figure 4:**
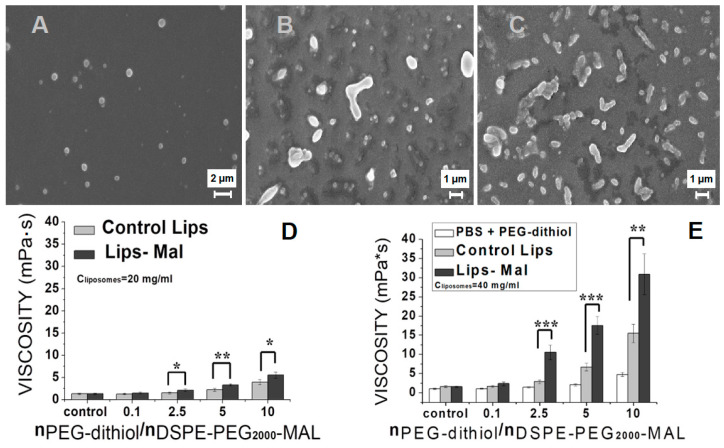
SEM images of PC-Lip-Mal (control liposomes) ((**A**); scale bar: 2 μm) and thioether cross-linked liposomes after the reaction of PC-Lip-Mal with PEG-dithiol ((**B**,**C**); scale bar: 1 μm). Viscosity (mPa^.^S) of liposome scaffold by the reaction of PEG-dithiol with PC-Lip-Mal, at 20 mg/mL (**D**) and 40 mg/mL (**E**). Asterisks show the significance of comparisons (* *p* ≤ 0.05, ** *p* ≤ 0.01, *** *p* ≤ 0.001).

Representative SEM images of PC-Lip-Mal before the reaction with PEG-dithiol (A) and after the reaction with PEG-dithiol (B and C) verify the cross-linking of liposomes, providing further evidence of the successful reaction of PEG-dithiols with the liposomal maleimide groups and the formation of nano-sized cross-linked liposomes (Figure 4A–C).

### 2.6. Effect of Thioether Cross-Linking on Liposome Scaffold Viscosity

Further studies regarding the effect of the reaction of PEG-dithiol with liposome maleimide groups on the liposome viscosity (which is also a parameter of a successful reaction) were performed. To this end, the reaction conditions were applied on Lips-Mal (PC/Chol/DSPE-PEG2000-maleimide (2:1:0.03) mol/mol), as well as control liposomes (PC/Chol/DSPE-PEG2000-OMe (2:1:0.03) mol/mol). Two liposome concentrations were used (20 and 40 mg/mL), while the excess of PEG-dithiol with respect to maleimide groups was adjusted to 0.1, 2.5, 5.0, and 10.0 mg/mL (according to our previous investigations). A control liposome dispersion was also used. The results of this experiment are shown in Figure 4D,E.

As can be seen, the reaction of PEG-dithiol with Lips-Mal of 20 mg/mL resulted in a low, however considerable, increase in liposome viscosity (Figure 4D), while a significantly higher increase in liposome viscosity was seen for the concentration of 40 mg/mL (Figure 4E). This was a clear effect of the reaction of PEG-dithiol with the surface-available maleimide groups, providing further evidence for the successful development of a nano-sized thioether cross-linked liposome scaffold.

### 2.7. Evaluation of Thioether Cross-Linked Liposomes as Drug Eluting Systems

In order to evaluate the potential of the synthesized cross-linked thioether type liposomes (abbreviated as LIP-di-thioether-PEG hereafter) to be used as sustained release drug delivery systems, their integrity in buffer phosphate (PBS) at pH 7.40 and fetal bovine serum (FBS) was evaluated.

It is well known that liposome integrity is decreased in the blood, due to liposome interaction with blood components, and their stability in the blood can be increased by increasing their membrane integrity, using more rigid lipids, the addition of cholesterol, and by coating liposome surface with PEG chains. Thus, in this set of experiments, PC lipid was replaced with hydrogenated PC (HPC) lipid, which is known to form more stable liposomes, while Chol and PEG were also used [47]. Furthermore, calcein was encapsulated into liposomes, as a method to evaluate liposomes’ integrity and calcein release, when liposomes are incubated with PBS and FBS. Calcein is a hydrophilic dye that is commonly used as a model of hydrophilic drugs, which can be encapsulated in liposomes to study their integrity during incubation in various media [48,49,50,51]. The calcein leakage method is used as a preferred method to evaluate the integrity of liposome types, due to the fact that calcein is entrapped in the vesicles at a concentration where its fluorescence is quenched, and FI is increased as calcein leaks out of the vesicle (and is diluted in the liposome dispersion media) [48,49,50,51].

The following calcein-encapsulated liposome compositions were prepared: (a) HPC/Chol/DSPE-PEG2000-OMe (2:1:0.03) mol/mol liposomes (control liposomes) and (b) HPC/Chol/DSPE-PEG2000-Mal liposomes (2:1:0.03) mol/mol (abbreviated as HPC-Lip-Mal hereafter). The lipid concentration of control liposomes and HPC-Lip-Mal was set at 13.5 and 14.0 mg/mL (close to the optimal lipid concentration of the reaction of PEG-dithiol with PC-Lip-Mal). Next, calcein liposomes (control and HPC-Lip-Mal) were reacted with PEG-dithiol, under the optimal reaction conditions (2.5 molar excess, 2 h, 27 °C). For this preparation, the initially formed calcein containing liposomes maleimides (purified from non-encapsulated calcein) were reacted with PEG-dithiol. During this reaction, no loss of efficacy is expected, since the drug (in our case, the drug model calcein) has already been encapsulated in liposomes, and the reaction of the high molecular PEG-dithiol is performed with the surface-available maleimide groups of the pre-formed liposomes. We report that, during the reaction, no calcein leakage was detected, which ensures efficient surface modification without any destruction of liposomes.

Liposome hydrodynamic diameter and polydispersity index (for both liposome compositions), before and after their reaction with PEG-dithiol, were measured (Table 3). As can be seen, the hydrodynamic diameter and polydispersity index of liposomes (control and HPC-Lip-Mal), before and after their reaction with PEG-dithiol, are consistent with our findings for PC-Lip-Mal liposomes. In brief, the size of HPC-Lip-Mal was increased from 100.3 nm to 145.7 nm (about 50% increment), whereas the size of control liposomes did not change (99.4 nm vs. 100.8 nm).

The polydispersity index of the cross-linked liposomes was also increased [from 0.199 (before reaction) to 0.317 (after reaction)]. Regarding the PDI values, although the PDI value of HPC-Lip-Mal is higher (0.317) than the corresponding after-reaction PDI value of PC-LIP-Mal (0.199), the liposome sample is still homogeneous (in the sense that no aggregates were present in the sample), as can be seen by the size distribution curves (Supplementary File, Figure S27). The ζ-potential values ranged between −2.20 and −2.65 mV for all liposomes (similar values were seen for the PC-Lip-Mal-reacted liposomes).

**Figure 5:**
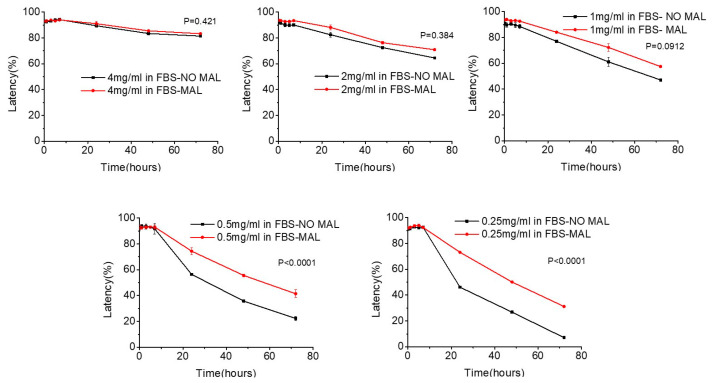
The % latency of calcein by the incubation of control liposomes (HPC-Lip) and thioether cross-linked liposomes (HPC-LIP-di-thioether-PEG) at different concentrations (4 mg/mL, 2 mg/mL, 1 mg/mL, 0.5 mg/mL, 0.25 mg/mL) in FBS at 37 °C.

Regarding the integrity of liposomes, this was investigated by measuring the % latency of calcein encapsulating liposomes during their incubation in buffer phosphate (PBS) pH 7.40 and fetal bovine serum (FBS) at 37 °C. The cross-linked liposomes, which were formed by the reaction of HPC-Lip-Mal with PEG-dithiol, and the corresponding control liposomes (without maleimide groups/cross-linking) were used. The liposome concentrations that were studied, as determined by an initial optimization process, were fixed at 4 mg/mL, 2 mg/mL, 1 mg/mL, 0.5 mg/mL, and 0.25 mg/mL. The results showed that, in the presence of PBS pH 7.40, all liposome structures, control, and cross-linked liposomes were stable (up to 72 h) for all tested concentrations with % latency values >95% (in all cases), indicating high stability of the liposomal scaffolds (analytical graphs of liposome stability in PBS are shown in the Supplementary File, Figure S28). On the contrary, in the presence of FBS, both types of liposomes (control and cross-linked) showed an increase in the released calcein as lipid concentration was lowered (from 4 to 0.25 mg/mL), indicating the lower stability of the liposomal scaffold. It is noteworthy that calcein release was higher for control liposomes than the corresponding cross-linked liposomes, at all time points (up to 72 h) (Figure 5).

It is apparent that the cross-linked liposome scaffolds behaved differently than the corresponding control liposomes by exhibiting higher stability (higher % latency) in the presence of FBS as a result of their cross-linking after the reaction of the liposome surface with the PEG-dithiol, obviously resulting in a more rigid liposome scaffold. This effect becomes obvious at lower concentrations, where chemical modification of liposomes is more significantly involved in liposome stability since fewer vesicles are incubated in the same amount of serum (and, consequently, of the serum components that are responsible for liposome membrane destabilization).

## 3. Materials and Methods

### 3.1. General Information

#### 3.1.1. Synthesis of α,ω-bis-Mercaptoacyl Poly(alkyl oxide)s

Poly(ethylene glycol) (PEG) with average molecular weight of 1.000 (950–1.050), 4.000 (3.500–4.500), 10.000 (9.000–12.500), and Poly(ethylene glycol)–block–poly(propylene glycol)–block–poly(ethylene glycol) (pluronic F127) with an average MW of 12.600 were purchased from Sigma-Aldrich (Darmstadt, Germany). In addition, 2-mercaptopropionic acid and 3-mercaptopropionic acid, as well as the reagents *N*,*N*′-diisopropylcarbodiimide (DIC), 4-(dimethylamino)pyridine (DMAP), trifluoroacetic acid (TFA), and triethylsilane (TES), were also purchased from Sigma-Aldrich (Darmstadt, Germany). 4-methoxytrityl chloride (Mmt-Cl) was gifted from CBL Patras S.A. (Patras, Greece). All other chemicals were purchased from Sigma-Aldrich (Darmstadt, Germany). All solvents were of analytical or HPLC grade and were purchased from Merck (Darmstadt, Germany).

Thin layer chromatography (TLC) was performed on silica gel 60 F254 plates (Merck, Darmstadt, Germany) and spot detection was carried out by UV light, and by charring with an aqueous solution of K_2_CO_3_/KMnO_4_. Flash column chromatography was performed on silica gel 230–400 mesh (Merck, Darmstadt, Germany). HPLC analysis was performed on a LiChrospher 100, RP-18, 250–4, 5 μm column, using acetonitrile (AcCN) and water (H_2_O) in the presence of 0.08% TFA as mobile phases. ESI-MS spectra were recorded on a Micromass Platform L.C. (Manchester, UK) at 30 eV. NMR spectra of the synthesized compounds were obtained at 600 MHz on a Bruker Avance III HD spectrometer. NMR spectra of functionalized liposomes and real-time monitoring of the reaction of PEG-dithiols with the functionalized liposomes were recorded at 27 °C on a Bruker Avance III high-definition four-channel 700 MHz NMR spectrometer (Billerica, MA, USA) equipped with a cryogenically cooled 5 mm ^1^H/^13^C/^15^N/D Z-gradient probe (TCI). Chemical shifts (*δ*) were referenced to the corresponding solvent peaks and are reported in ppm.

#### 3.1.2. Liposome Preparations

Egg Phosphatidylcholine (PC), Phosphatidylcholine, hydrogenated (HPC), 1,2-distearoyl-sn-glycero-3-phosphoethanolamine-*N*-[maleimide(polyethylene glycol)-2000] (ammonium salt) (DSPE-PEG2000-Mal), and 1,2-distearoyl-sn-glycero-3-phosphoethanolamine-*N*-[methoxy(polyethylene glycol)-2000] (ammonium salt) (DSPE-PEG2000-OMe) were purchased from Lipoid, Germany. Cholesterol (Chol), Calcein, Triton X-100, Sephadex G-50, and Sepharose CL-4B were purchased from Sigma-Aldrich (Darmstadt, Germany). Fetal bovine serum (FBS) was obtained from Biosera (Nuaille, France). All solvents used were of analytical or HPLC grade and were purchased from Merck (Darmstadt, Germany). Any other chemicals used were purchased from Sigma-Aldrich (Darmstadt, Germany).

For the liposome preparation, a bath sonicator (Branson 2510, Thermo Fisher Scientific, Waltham, MA, USA) or a microtip-probe high-intensity sonicator (Sonics and Materials, Inc., VC750, Newtown, CT, USA) was used. For liposome purification, size-exclusion chromatography was performed on Sepharose CL-4B (Sigma-Aldrich, Darmstadt, Germany). A UVmini-1240 spectrophotometer was used for lipid concentration measurement (Shimadzu, Kyoto, Japan). A Shimadzu RF-5301PC spectrofluorometer (Shimadzu, Kyoto, Japan) was used for the measurement of the fluorescence intensity (FI) of calcein in samples (EX/EM 490 nm/525 nm; 5 nm slits).

#### 3.1.3. Liposome Physicochemical Characterization

The mean hydrodynamic diameter and the polydispersity index (PDI) of the vesicles (dispersed in PBS at 0.2 mg/mL phospholipid concentration) was measured by dynamic light scattering (DLS) at 25 °C (173° angle) on a Malvern Nano-ZS (Malvern Instruments, Worcestershire, UK). Zeta potential (ζ-potential) values were measured also at 25 °C in the same samples by Doppler electrophoresis. Viscosity was recorded on a rolling-ball viscometer Lovis 2000 M/ME (Anton Paar GmbH, Graz, Austria).

### 3.2. Synthetic Procedures

#### 3.2.1. *S*-Mmt-Mercaptopropionic Acids (*S*-Mmt-2-Mercaptopropionic Acid **6a**; *S*-Mmt-3-Mercaptopropionic Acid **6b**)

4-methoxytrityl chloride (**7**; 2.5 g; 8.1 mmol) was dissolved in DCM (5.5 mL) and, to this solution, 2-mercaptopropionic acid (**8a**; 7.36 mmol; 0.65 mL) or 3-mercaptopropionic acid (**8b**; 7.36 mmol; 0.64 mL) was added and the reaction mixture was stirred at room temperature for about 30 min (the reaction progress was monitored by TLC). The reaction mixture was then concentrated on a rotary evaporator under reduced pressure and the oily product that was formed was diluted with ethyl acetate (EtOAc) and washed with water (×3). The organic layer was collected and further dried over sodium sulfate and the filtrate was concentrated until the formation of an oily residue. To this, diethyl ether (DEE) was added (10 mL), and the resulting solution was placed at 4 °C (overnight). The white crystals that were formed were finally filtered and washed with DEE (×3) (3 × 10 mL) to afford **6a** and **6b** as white solids: **6a** (2.56 g; yield: 92%); m.p. (153–155 °C); **6b** (2.50 g; yield 90%); m.p. 137–138 °C; **6a:** ^1^H-NMR (methanol-*d*_4_, 600 MHz) *δ* 7.43 (d, *J* = 8.2 Hz, 4H, H-Ar), 7.33 (d, *J* = 8.9 Hz, 2H. H-Ar); 7.28 (t, *J* = 7.2 Hz, 4H, H-Ar); 7.22 (t, *J* = 7.0 Hz, 2H. H-Ar), 6.85 (d, *J* = 8.9 Hz, 2H, H-Ar), 3.79 (s, 3H, H-c), 2.92 (q, *J* = 7.3 Hz, 1H, H-b), 1.05 (d, *J* = 7.3 Hz, 3H, H-a); ^13^C-NMR (CDCl_3_, 150 MHz) *δ* 178.59 (C-e), 158.28, 144.48, 136.20, 130.87, 129.48, 127.96, 126.85, 113.21 (C-Ar), 67.88 (C-d), 55.22 (C-c), 42.43 (C-b), 18.58 (C-a); **6b:** ^1^H-NMR (methanol-*d*_4_, 600 MHz) *δ* 7.38 (d, *J* = 7.9 Hz, 4H, H-Ar), 7.31–7.23 (m, 6H, H-Ar), 7.20 (t, *J* = 7.2 Hz, 2H, H-Ar), 6.84 (d, *J* = 8.7 Hz, 2H, H-Ar), 3.78 (s, 3H, H-c), 2.40 (t, *J* = 7.2 Hz, 2H, H-a), 2.20 (t, *J* = 7.2 Hz, 2H, H-b); ^13^C-NMR (CDCl_3_, 150 MHz) *δ* 176.87 (C-e), 158.12, 144.86, 136.63, 130.74, 129.45, 127.91, 126.66, 113.19 (C-Ar), 66.44 (C-d), 55.23 (C-c), 33.24 (C-b), 26.55 (C-a).

#### 3.2.2. di-*S*-Mmt-di-2-Mercaptopropionyl PEG10.000 (**14**)

First, **6a** (0.40 g; 1.05 mmol) and DMAP (0.14 g; 1.155 mmol) were placed into a round bottom flask and completely dissolved in DCM (4 mL). DIC (1.155 mmol; 0.18 mL) was then added and the resulting mixture was stirred for 5 min at room temperature. The activated **6a** was then transferred to another flask where PEG10.000 (5.0 g, 0.5 mmol) had been dissolved in DCM (8 mL), and the resulting reaction mixture was stirred overnight at room temperature. The reaction progress was monitored by TLC, where the disappearance of the starting material (PEG) and the formation of one product (**14**) was clear evidence of the reaction progress/completion. The reaction mixture was then filtered, and the filtrate was further extracted with DCM and 10% citric acid. The two layers were separated, and the organic layer was further washed with water (×2). Finally, the organic layer was dried over magnesium sulfate and the filtrate was concentrated under reduced pressure until the formation of an oily residue. To this, EtOAc (25 mL) was added, until the oil was dissolved, and then DEE was slowly added (25 mL) and the solution was allowed to stand at room temperature until the formation of white crystals. Then, the solution was placed at 4 °C to allow complete crystallization (overnight) and the white crystals that were formed were filtered and washed with a mixture of EtOAc:DEE (1:1) (×1) (40 mL) and DEE (×2) (2 × 40 mL) and then dried over P_2_O_5_ to afford **14** as a white solid (4.96 g; yield: 92.5%); m.p.: 55–57 °C; ^1^H-NMR (methanol-*d*_4_, 600 MHz) *δ* 7.42 (d, *J* = 7.6 Hz, 8H, H-Ar), 7.37–7.26 (m, 12H, H-Ar), 7.22 (t, *J* = 7.2 Hz, 4H, H-Ar), 6.86 (d, *J* = 8.7 Hz, 4H, H-Ar), 4.12–4.08 (m, 2H, H-c), 3.97–3.93 (m, 2H, H-c), 3.79 (s, 6H, H-g), 3.77–3.46 (H-d,e,f), 2.95 (q, *J* = 7.2 Hz, 2H, H-b), 1.12 (d, *J* = 7.3 Hz, 6H, H-a); ^13^C-NMR (CDCl_3_, 150 MHz) *δ* 173.54 (C-i), 158.15, 144.68, 144.56, 136.36, 130.88, 129.54, 129.48, 127.86, 126.69, 113.11 (C-Ar), 70.54, 68.74 (C-d,e,f), 67.67 (C-h), 64.04 (C-c), 55.20 (C-g), 42.33 (C-b), 18.67 (C-a).

#### 3.2.3. di-2-Mercaptopropionyl PEG10.000 (**17**)

A mixture of 5% TFA in DCM/TES (95:5) (80 mL) was added into a round flask containing **14** (4.0 g; 37.31 × 10^–2^ mmol) and the mixture was stirred at room temperature, where a gradual discoloration of the cleavage mixture was observed. Complete/irreversible cleavage was confirmed by adding a few drops of concentrated TFA until no color reappeared. TLC was also indicative of a complete/irreversible cleavage of *S*-Mmt groups. Then, the cleavage mixture was concentrated under reduced pressure, and the resulting oily product was treated with an excess of MeOH and concentrated again (twice). To the oily product that was finally formed, DEE (40 mL) was added, and the mixture was left at room temperature until precipitation began and then it was placed at 4 °C to allow complete precipitation. The solid was finally filtered and washed twice with DEE (2 × 40 mL) and dried over P_2_O_5_ to afford **17** as a white solid (3.26 g; yield: 86%); m.p.: 57–58 °C; ^1^H-NMR (CDCl_3_, 600 MHz) *δ* 4.30–4.26 (m, 4H, H-c), 3.77–3.49 (H-b, d, e, f), 2.19 (d, 2H, *J* = 8.3 Hz, SH), 1.52 (d, *J* = 7.0Hz, 6H, H-a); ^13^C-NMR (CDCl_3_, 150 MHz) *δ* 173.69 (C-g), 70.54, 68.91 (C-d, e, f), 64.44 (C-c), 35.54 (C-b), 20.97 (C-a).

#### 3.2.4. di-*S*-Mmt-di-2-Mercaptopropionyl PEG4.000 (**15**)

First, **6a** (0.2 g; 52.50 × 10^–2^ mmol) and DMAP (70.55 mg; 57.75 × 10^–2^ mmol) were completely dissolved in DCM (4 mL). DIC (57.75 × 10^–2^ mmol; 90.42 μL) was added and the mixture was stirred for 5 min at room temperature. The activated **6a** was then added to another flask where PEG4.000 (1.0 g, 0.25 mmol) had been dissolved in DCM (3 mL), and the resulting mixture was stirred overnight at room temperature. The reaction progress was monitored by TLC, where the disappearance of the starting material (PEG) and the formation of one product (**15**) was clear evidence of the reaction progress/completion. The reaction mixture was then filtered, and the filtrate was further extracted in DCM and 10% citric acid. The two layers were separated, and the organic layer was further washed with water (×2). Finally, the organic layer was dried over magnesium sulfate and the filtrate was concentrated under reduced pressure until the formation of an oily residue. This was further crystallized in boiling ethanol (EtOH) (12 mL) and the crystalline product (after overnight precipitation at 4 °C) was filtered at 4 °C (and further recrystallized in EtOH). The crystals were collected and dried over P_2_O_5_ to afford **15** as a white solid (1.05 g; yield: 94%); m.p.: 47–48 °C; ^1^H-NMR (CDCl_3_, 600 MHz) *δ* 7.43 (d, *J* = 8.1 Hz, 8H), 7.33 (d, *J* = 8.8 Hz, 4H. H-Ar), 7.26 (t, *J* = 7.6 Hz, 8H) + CDCl_3_, 7.19 (t, *J* = 7.3 Hz, 4H, H-Ar), 6.80 (d, *J* = 8.8 Hz, 4H, H-Ar), 4.13–4.08 (m, 2H, H-c), 3.98–3.93 (m, 2H, H-c), 3.78 (s, 6H, H-g), 3.77–3.50 (H-d,e,f), 2.98 (q, *J* = 7.3 Hz, 2H, H-b), 1.16 (d, *J* = 7.3 Hz, 6H, H-a); ^13^C-NMR (CDCl_3_, 150 MHz) *δ* 173.55 (C-i), 158.16, 144.68, 144.56, 136.36, 130.88, 129.54, 129.48, 127.86, 126.69, 113.11 (C-Ar), 70.54, 68.74 (C-d,e,f), 67.67 (C-h), 64.04 (C-c), 55.20 (C-g), 42.33 (C-b), 18.67 (C-a).

#### 3.2.5. di-2-Mercaptopropionyl PEG4.000 (**18**)

A mixture of 5% TFA in DCM/TES (95:5) (10 mL) was added into a round flask containing **15** (0.5 g; 10.59 × 10^–2^ mmol), and the mixture was stirred at room temperature, where a gradual discoloration of the cleavage mixture was observed. Complete/irreversible cleavage was confirmed by adding a few drops of concentrated TFA until no color reappeared. TLC was also indicative of a complete/irreversible cleavage of *S*-Mmt groups. Then, the cleavage mixture was concentrated under reduced pressure, and the resulting oily product was treated with an excess of MeOH and concentrated again (twice). To the oily product that was formed, DEE was added (8 mL), and the mixture was left at room temperature until precipitation started and then it was placed at 4 °C to allow for complete precipitation. The precipitate was finally filtered, washed with DEE (×2) (2 × 8 mL) and further dried over P_2_O_5_ to afford **18** as a white solid (0.41 g; yield: 93%); m.p.: 50–51.5 °C; ^1^H-NMR (CDCl_3_ 600 MHz) *δ* 4.29–4.26 (m, 4H, H-c), 3.77–3.49 (H-b, d, e, f), 2.19 (d, 2H, *J* = 8.3 Hz, SH), 1.51 (d, *J* = 7.0 Hz, 6H, H-a); ^13^C-NMR (CDCl_3_, 150 MHz) *δ* 173.70 (C-g), 70.55, 68.91 (C-d, e, f), 64.44 (C-c), 35.54 (C-b), 20.97 (C-a).

#### 3.2.6. di-*S*-Mmt-di-2-Mercaptopropionyl PEG1.000 (**16**)

First, **6a** (1.6 g; 4.2 mmol) and DMAP (0.56 g; 4.6 mmol) were completely dissolved in DCM (4 mL). DIC (4.6 mmol; 0.72 mL) was added and the mixture was stirred for 5 min at room temperature. The activated **6a** was then transferred to another flask where PEG1.000 (2.0 g; 2.0 mmol) had been dissolved in DCM (3 mL), and the reaction mixture was stirred overnight at room temperature. The reaction progress was monitored by TLC, where the disappearance of the starting material (PEG1.000) and the formation of one product (**16**) was clear evidence of the reaction progress/completion. The reaction mixture was then filtered, and the filtrate was further extracted in DCM and 10% citric acid. The two layers were separated, and the organic layer was further washed with water (×2). Finally, the organic layer was dried over magnesium sulfate and the filtrate was concentrated under reduced pressure until the formation of **16** as an oily residue, which was directly used in the next step.

#### 3.2.7. di-2-Mercaptopropionyl PEG1.000 (**19**)

A mixture of 5% TFA in DCM/TES (95:5) (80 mL) was poured into the round flask containing **16**, and the mixture was stirred at room temperature, where a gradual discoloration of the cleavage mixture was observed. Complete/irreversible cleavage of Mmt groups was confirmed by adding a few drops of concentrated TFA until no color reappeared. TLC analysis was also indicative of a complete/irreversible cleavage of *S*-Mmt groups. Then, the cleavage mixture was concentrated, and the resulting oily product that was formed was treated with an excess of MeOH and evaporated again (twice). The resulting oily product was washed several times with hexane (Hex) (5 × 20 mL) and, finally, dried over P_2_O_5_ to afford **19** as oil (2.1 g; yield: 89% over two steps); ^1^H-NMR (CDCl_3_ 600 MHz) *δ* 4.25–4.31 (m, 4H, H-c), 3.80–3.49 (H-b, d, e, f), 2.19 (d, 2H, *J* = 8.3 Hz, SH), 1.51 (d, 6H, *J* = 7.0 Hz, H-a); ^13^C-NMR (CDCl_3_, 150 MHz) *δ* 173.70 (C-g), 70.55, 68.91 (C-d, e, f), 64.44 (C-c), 35.54 (C-b), 20.97 (C-a).

#### 3.2.8. di-*S*-Mmt-di-3-Mercaptopropionyl Pluronic (**20**)

First, **6b** (0.126 g; 33.33 × 10^–2^ mmol) and DMAP (44.8 mg; 36.67 × 10^–2^ mmol) were completely dissolved in DCM (0.5 mL). DIC (36.67 × 10^–2^ mmol; 57.4 μL) was then added and the mixture was stirred for 5 min at room temperature. The activated **6b** was then transferred to another flask where pluronic (2.0 g; 15.87 × 10^–2^ mmol) had been dissolved in DCM (3 mL), and the reaction mixture was stirred overnight at room temperature. The reaction progress was monitored by TLC analysis, where the disappearance of the starting material (pluronic) and the formation of one product (**20**) was clear evidence of the reaction progress/completion. Then, the reaction mixture was filtered, and the filtrate was extracted in DCM and 10% citric acid. The two layers were separated, and the organic layer was further washed with water (×2). The organic layer was dried over magnesium sulfate and filtered, and the filtrate was concentrated until the formation of an oily residue. To this, DEE (40 mL) was added, and the white precipitate that was formed was filtered and further washed with DEE (×3) (3 × 40 mL), and, finally, dried over P_2_O_5_ to afford **20** as a white solid (1.97 g; yield: 93%); m.p.: 48.5–50 °C; ^1^H-NMR (CDCl_3_, 600 MHz) *δ* 7.38 (d, *J* = 8.0 Hz, 8H, H-Ar), 7.30–7.22 (m, 12H, H-Ar)+CDCl_3_, 7.18 (t, *J* = 7.2 Hz, 4H, H-Ar), 6.79 (d, *J* = 8.6 Hz, 4H, H-Ar), 4.19–4.14 (m, 4H, H-c), 3.77 (s, 6H, H-j), 3.75–3.58 (H-d, e, f), 3.57–3.48 (m, 130H, H-h), 3.41–3.33 (m, 65H, H-g), 2.43 (t, *J* = 7.3 Hz, 4H, H-a), 2.27 (t, *J* = 7.3 Hz, 4H, H-b), 1.14–1.09 (m, 195H, H-i); ^13^C-NMR (CDCl_3_, 150 MHz) *δ* 171.82 (C-l), 158.07, 144.94, 136.69, 130.74, 129.45, 127.87, 126.60, 113.15 (C-Ar), 75.50, 75.35, 75.30, 75.10, 73.34, 72.94, 72.89, 72.85, 72.80, 70.82, 70.54, 69.02, 68.49, 63.72 (C-PEO, C-PPO), 68.49 (C-k), 55.20 (C-j), 33.45 (C-b), 26.86 (C-a), 17.43, 17.30 (C-i).

#### 3.2.9. di-3-Mercaptopropionyl Pluronic (**21**)

A mixture of 5% TFA in DCM/TES (95:5) (26.5 mL) was added into a round flask containing **20** and the mixture was stirred at room temperature, where a gradual discoloration of the cleavage mixture was observed. Complete/irreversible cleavage of Mmt groups was confirmed by adding a few drops of concentrated TFA until no color reappeared. TLC was also indicative of a complete/irreversible cleavage of *S*-Mmt groups. Then, the cleavage mixture was concentrated under reduced pressure, and the resulting oily product was treated with an excess of MeOH and concentrated again (twice). To the oily product that was formed, DEE (30 mL) was added, until the formation of a white solid, and the suspension was left to stand at room temperature, overnight, to allow complete precipitation. The precipitate was finally filtered, washed with DEE (×3) (3 × 30 mL) and dried over P_2_O_5_ to afford **21** as a white solid (1.85 g; yield: 91% over two steps); m.p.: 51–52.5 °C; ^1^H-NMR (CDCl_3_, 600 MHz) *δ* 4.26–4.22 (m, 4H, H-c), 3.76–3.57 (H-d, e, f), 3.56–3.43 (m, 130H, H-h), 3.41–3.31 (m, H65, H-g), 2.78–2.72 (m, 4H, H-a), 2.68–2.62 (m, 4H, H-b), 1.66 (t, 2H, *J* = 8.3 Hz, SH), 1.16–1.05 (m, 195H, H-i); ^13^C-NMR (CDCl_3_, 150 MHz) *δ* 171.62 (C-j), 75.50, 75.34, 75.30, 75.10, 73.34, 72.94, 72.90, 72.85, 72.81, 72,56, 70.82, 70.54, 70.29, 69.02, 68.58, 68.49, 63.87, 61.69 (C-c, d, e, f, g, h), 33.99 (C-b), 32.99 (C-a), 17.43, 17.31 (C-i).

#### 3.2.10. Liposome Preparation Procedure

Small unilamellar vesicle (SUV)-type liposomes were prepared by the thin film hydration method and high-intensity sonication. In brief, the appropriate amounts of lipids (PC or HPC; Chol; DPSE-PEG2000-Mal or DSPE-PEG2000-OMe) in order to have the desired mol ratio (2:1:0.03) were dissolved in a chloroform (CHCl_3_)/MeOH (2:1 *v*/*v*) mixture. In a typical preparation, PC (50.0 mg; 65.10 μmol), Chol (12.59 mg; 32.55 μmol), DSPE-PEG2000-Mal (2.87 mg; 97.66 × 10^–2^ μmol) or DSPE-PEG-OMe (2.74; 97.66 × 10^–2^ μmol) were mixed and the resulting mixture was evaporated under reduced pressure until the formation of a thin film lipid layer. The lipid film was treated with N_2_ gas and was subsequently connected to a vacuum pump for 12 h in order to remove any traces of organic solvent. The lipid film was next hydrated at the desired lipid concentration of 20 mg/mL (i.e., 2.5 mL) with D_2_O (in case of liposomes intended for 1D CPMG ^1^H-NMR) or PBS pH 7.40 at room temperature—in case of PC-containing liposomes—or at 60 °C for HPC-containing liposomes. For calcein encapsulating liposomes, a 100 mM solution of calcein, prepared in the same buffer, was used for thin film hydration. After complete lipid hydration and formation of multilamellar vesicle (MLV) type liposomes, the liposome dispersion was placed under the microtip of a probe sonicator for 10 min, or until the liposome dispersion was completely clear. The liposome dispersion was then allowed to stand for 1 h at a temperature above the lipid transition temperature to anneal any structural defects. The calcein-encapsulating liposomes were purified from non-entrapped calcein by size-exclusion chromatography on a Sepharose 4B-CL column that was eluted with PBS pH 7.40. The Stewart assay, a colorimetric method that is routinely used for the quantification of phospholipids, was used for the measurement of the final phospholipid content/concentration of liposomes [52].

#### 3.2.11. Reaction of di-Thioether-PEGs with Pre-Formed LIP-Maleimides

Pre-formed liposomes maleimide of the appropriate lipid concentration were incubated with PEG-dithiol at 27 °C, either in D_2_O (in the case of liposomes intended for 1D CPMG ^1^H-NMR), or PBS pH 7.40. In a typical reaction, PC/Chol/DSPE-PEG-Mal (2:1:0.03) liposomes adjusted at 20 mg/mL lipid concentration with the Stewart assay, therefore, consisted of PC (20 mg; 26.04 μmol), Chol (5.03 mg; 13.02 μmol), DSPE-PEG-Mal (1.15 mg; 39.06 × 10^–2^ μmol) and were mixed with di-2-mercaptopropionyl PEG10.000 **17** (2.5 molar excess in respect to lipid-maleimide; 9.94 mg; 97.66 × 10^–2^ μmol), and the reaction mixture was incubated at 27 °C for 2 h. The obtained cross-linked thioether liposome scaffolds were further purified from the non-reacted PEG-dithiol with size-exclusion chromatography on a Sepharose 4B-CL column eluted with PBS pH 7.40 and the final liposome concentration was measured by the Stewart assay [52].

#### 3.2.12. Liposome Integrity Studies

To monitor the integrity of liposomes (control and after their reaction with PEG-dithiol), calcein-encapsulating liposomes (4 mg/mL, 2 mg/mL, 1 mg/mL, 0.5 mg/mL, 0.25 mg/mL phospholipid) were prepared. Calcein was encapsulated in liposomes at 100 mM concentration (where calcein fluorescence is quenched), and liposomes were incubated at 37 °C in PBS pH 7.40, as well as in FCS (80% *v*/*v*). Calcein latency (%) values were calculated at various time points by taking samples from the incubated liposomes and measuring calcein FI. In brief, at each time point, 20 μL of vesicle dispersions were taken and diluted in 4 mL of PBS buffer. The FI was measured (EX 470 nm, EM 520 nm) before and after the addition of Triton X-100 at a final concentration of 1% *v*/*v*, which ensures complete liposome disruption and release of all encapsulated (and latent) dye. Latency (%) was calculated from the equation Latency (%) = 100 × [(1.1 × Fat) − Fbt]/(1.1 × Fat), where Fbt and Fat are calcein fluorescence intensities before and after the addition of Triton X-100, respectively (for fat multiplication by 1.1 was applied for correction due to dilution).

#### 3.2.13. Scanning Electron Microscopy (SEM)

The vesicular morphology of the liposome preparations was recorded using SEM. SEM images of PC-Lip-Mal before their reaction with PEG-dithiol and after their reaction with PEG-dithiol were recorded. In brief, 100 μL of each sample were placed on clean specimen mounts, which were dried overnight first in a fume hood and then under vacuum (2.25 Pa). Subsequently, the samples were sputtered with gold and observed in a JEOL JSM-35 (Akishima, Japan) operating at an acceleration voltage of 25 kV with 0° tilt (×500).

## 4. Conclusions

A new approach for the simple and efficient synthesis of α,ω-homobifunctional mercaptoacyl poly(alkyl oxide)s has been proposed. This method is based on the esterification of suitably *S*-Mmt-protected mercapto acids with the hydroxyl end groups, by the use of DIC and DMAP as the carboxylic acid activating system. The advantages of this approach are summarized below: (a) The use of commercially available or synthesized *S*-Mmt mercapto acids allows structural diversity of the added mercaptoacyl groups. (b) Previously reported methods make use of high excess of the mercapto acid (usually a ×10 to ×20 times excess), as well as relatively harsh reaction conditions and specially designed glassware (reflux conditions and Dean–Stark trap). In our approach, an equimolar amount of the *S*-Mmt-mercapto acid with respect to the available hydroxyl groups is used and the reaction is performed at room temperature by the use of simple esterification reagents. In addition, no reducing agents and/or inert conditions are required, minimizing the necessary glassware to a single flask. Moreover, there are no unpleasant odors during the reaction or workup/purification procedures (usually encountered in the presence of thiols). (c) Protection of the thiol groups during the esterification reaction ensures that no by-products are formed during the reaction process, while cleavage of the labile *S*-Mmt group can be achieved fast and irreversibly in mild acidic conditions. (d) The applied conditions allow quantitative functionalization of both α,ω-hydroxyls. (e) The *S*-Mmt-protected poly(alkyl oxide)s can be stored until the free thiol compounds are needed, avoiding the formation of any oxidation by-products during long-term storage. According to our knowledge, this is the first time that *S*-Mmt-marcapto acids were used for the synthesis of α,ω-homobifunctional mercaptoacylated poly(alkyl oxide)s, while the applied methods allow an easy and efficient synthesis of the title compounds.

The synthesized dithiols were further used as cross-linking agents for the synthesis of a novel biomacromolecular nano-sized liposome scaffold with the reaction of pre-formed liposomes bearing maleimide groups on their surface with PEG-dithiol, and the reaction progress was followed by 1D CPMG ^1^H-NMR. According to our knowledge, this is the first time that ^1^H-NMR CPMG is used for the real-time monitoring of the reaction between surface-available nano-sized particle functionalities and organic compounds of any type, including PEG-dithiol macromolecules.

The liposomal scaffold that was synthesized is the third novelty of this work. According to our knowledge, this is the first cross-linked liposomal scaffold that has been synthesized to date, which maintains the nano characteristics of the initial liposomes. This scaffold also allowed for the sustained release of a hydrophilic dye that is commonly used as a hydrophilic drug model (calcein) providing evidence for the efficient synthesis of an innovating nano-sized liposome scaffold for sustained release of drugs.

## Figures and Tables

**Table 1:** Mean hydrodynamic diameter (nm) and polydispersity index (PDI) for control liposomes (PC/Chol/DSPE-PEG2000-OMe (2:1:0.03 mol/mol)) and Lips-Mal (PC/Chol/DSPE-PEG2000-Mal (2:1:0.03 mol/mol)) before and after their reaction with PEG-dithiol, at several mol PEG-dithiol/mol maleimide ratios, and lipid concentration of 20 mg/mL ^a^.

		Control Lips + PEG-Dithiol		PC-Lip-Mal + PEG-Dithiol	
Sample No ^b^	mol PEG-Dithiol/mol Maleimide	Mean Hydrodynamic Diameter (nm)	PDI	Mean Hydrodynamic Diameter (nm)	PDI
1	-	105.10 ± 2.07	0.158	101.80 ± 0.47	0.190
2	2.5	96.48 ± 0.74	0.199	128.90 ± 1.3	0.199
3	5	111.60 ± 0.95	0.231	121.30 ± 1.4	0.318
4	10	105.50 ± 3.7	0.260	153.10 ± 10.4	0.409

^a^ The values reported are mean values from three measurements of three independent samples. ^b^ Sample 1 corresponds to liposomes (control and PC-Lip-Mal) at time zero (before the addition of PEG-dithiol); Samples 2–4 correspond to liposomes (control and PC-lip-Mal) treated with different excess of PEG-dithiol.

**Table 2:** Mean hydrodynamic diameter (nm) and polydispersity index (PDI) for control liposomes (PC/Chol/DSPE-PEG2000-OMe (2:1:0.03 mol/mol)) and Lips-Mal (PC/Chol/DSPE-PEG2000-maleimide (2:1:0.03 mol/mol)) before and after their reaction with PEG-dithiol, at several mol PEG-dithiol/mol maleimide ratios and lipid concentration of 40 mg/mL ^a^.

		Control Lips + PEG-Dithiol		PC-Lip-Mal + PEG-Dithiol	
Sample No ^b^	mol PEG-Dithiol/mol Maleimide	Mean Hydrodynamic Diameter (nm)	PDI	Mean Hydrodynamic Diameter (nm)	PDI
1	-	89.38 ± 0.85	0.154	90.93 ± 0.98	0.178
2	2.5	89.06 ± 0.79	0.169	138.30 ± 2.2	0.244
3	5	91.90 ± 1.6	0.246	140.20 ± 3.4	0.260
4	10	100.60 ± 1.4	0.261	148.00 ± 4.5	0.375

^a^ The values reported are mean values from three measurements of three independent samples. ^b^ Sample 1 corresponds to liposomes (control and PC-Lip-Mal) at time zero (before the addition of PEG-dithiol); Samples 2–4 correspond to liposomes (control and PC-lip-Mal) treated with different excesses of PEG-dithiol.

**Table 3:** Mean hydrodynamic diameter (nm), polydispersity index (PDI) and ζ-potential (mV) values for control liposomes (HPC/Chol/DSPE-PEG2000-OMe (2:1:0.03) mol/mol) and HPC-Lip-Mal (HPC/Chol/DSPE-PEG2000-maleimide (2:1:0.03) mol/mol) before and after their reaction with PEG-dithiol (2.5 molar excess) at lipid concentrations of 13.5 and 14.0 mg/mL.

Lipid Composition	Mean Hydrodynamic Diameter (nm)		PDI	
	Before Reaction	After Reaction	Before Reaction	After Reaction
Control Lips	99.4 ± 0.543	100.8 ± 0.757	0.180	0.192
HPC-Lip-Mal	100.3 ± 0.621	145.7 ± 2.875	0.199	0.317

## Data Availability

The data presented in this study are available on request from the corresponding authors.

## Supplementary Materials

The following supporting information can be downloaded at https://www.mdpi.com/article/10.3390/molecules29061312/s1, Figure S1: ^1^H-NMR of *S*-Mmt-2-mercaptopropionic acid (thiolactic acid) (**6a**) in methanol-*d*_4_; Figure S2: ^13^C-NMR of *S*-Mmt-2-mercaptopropionic acid (thiolactic acid) (**6a**) in CDCl_3_; Figure S3: ^1^H-NMR of *S*-Mmt-3-mercaptopropionic acid (**6b**) in methanol-*d*_4_; Figure S4: ^13^C-NMR of *S*-Mmt-3-mercaptopropionic acid (**6b**) in CDCl_3_; Figure S5: analytical hplc of *S*-Mmt-2-mercaptopropionic acid (thiolactic acid) (**6a**) (A1, A2) and *S*-Mmt-3-mercaptopropionic acid (**6b**) (B1, B2) at 214 and 265 nm; column: LiChrospher 100, RP-18, 250-4, 5 μm; mobile phase solvents: AcCN/water (0.08% TFA); gradient conditions: 20% to 100% AcCN in 30 min; Figure S6: ^1^H-NMR of di-*S*-Mmt-di-2-mercaptopropionyl PEG10.000 (**14**) in methanol-*d*_4_; Figure S7: ^13^C-NMR of di-*S*-Mmt-di-2-mercaptopropionyl PEG10.000 (**14**) in CDCl_3_; Figure S8: ^1^H-NMR of di-2-mercaptopropionyl PEG10.000 (**17**) in CDCl_3_; Figure S9: ^13^C-NMR of di-2-mercaptopropionyl PEG10.000 (**17**) in CDCl_3_; Figure S10: ^1^H-NMR of di-*S*-Mmt-2-mercaptopropionyl PEG4.000 (**15**) in CDCl_3_; Figure S11: ^13^C-NMR of di-*S*-Mmt-2-mercaptopropionyl PEG4.000 (**15**) in CDCl_3_; Figure S12: ^1^H-NMR of di-2-mercaptopropionyl PEG4.000 (**18**) in CDCl_3_; Figure S13: ^1^H-NMR of di-2-mercaptopropionyl PEG4.000 (**18**) in CDCl_3_; Figure S14: ^1^H-NMR of di-2-mercaptopropionyl PEG1.000 (**19**) in CDCl_3_; Figure S15: ^1^H-NMR of di-2-mercaptopropionyl PEG1.000 (**19**) in CDCl_3_; Figure S16: ESI-MS of di-2-mercaptopropionyl PEG10.000 (**17**); (M + 12H)/12; Figure S17: ESI-MS of di-2-mercaptopropionyl PEG4.000 (**18**); (M + 6H)/6; Figure S18: ESI-MS of di-2-mercaptopropionyl PEG1.000 (**19**); (M + 2H)/2, M + H; Figure S19: ^1^H-NMR of di-*S*-Mmt-di-3-mercaptopropionyl pluronic (**20**) in CDCl_3_; Figure S20: ^13^C-NMR of di-*S*-Mmt-di-3-mercaptopropionyl pluronic (**20**) in CDCl_3_; Figure S21: ^1^H-NMR of di-3-mercaptopropionyl pluronic (**21**) in CDCl_3_; Figure S22: ^13^C-NMR of di-3-mercaptopropionyl pluronic (**21**) in CDCl_3_; Figure S23: ESI-MS of di-3-mercaptopropionyl pluronic (**21**); M + 14H/14, M + 15H/15; Figure S24: ^1^H-NMR spectrum of PC/Chol/DSPE-PEG2000-Mal (2:1:0.03) mol/mol liposomes in D_2_O using CPMG pulse sequence at 700 MHz, T = 27 °C. The lipid concentration was adjusted at 20 mg/mL (in D_2_O); Figure S25: ^1^H-NMR analysis of PC/Chol/DSPE-PEG2000-Mal (2:1:0.03) mol/mol liposomes in D_2_O using CPMG pulse sequence at 700 MHz, T = 27 °C, at t = 0, 45, 75, 180 min. The lipid concentration was adjusted at 20 mg/mL (in D_2_O); Figure S26: real-time monitoring of the reaction progress between pre-formed PC-Lip-Mal and PEG-dithiol in D_2_O using ^1^H-NMR CPMG pulse sequence at 700 MHz, T = 27 °C. The lipid concentration was adjusted at 20 mg/mL (in D_2_O); Figure S27: size distribution before (A) and after reaction (B) for control liposomes. Size distribution before (C) and after reaction (D) for Lips-Mal; Figure S28: % latency of calcein by the incubation of liposomes in PBS pH 7.40 at 37 °C. Control liposomes (HPC-Lip; PBS-NO MAL) and thioether cross-linked liposomes (HPC-LIP-di-thioether-PEG; PBS-MAL) at different concentrations (4 mg/mL, 2 mg/mL, 1 mg/mL, 0.5 mg/mL, 0.25 mg/mL).
