# Supplementary material for: Synthesis of α,ω-bis-Mercaptoacyl Poly(alkyl oxide)s and Development of Thioether Cross-Linked Liposome Scaffolds for Sustained Release of Drugs
Source: Molecules. 2024 Mar 15;29(6):1312. doi: 10.3390/molecules29061312 (PMC10975287; doi:10.3390/molecules29061312)
Supplement: Supplementary file 1 [file molecules-29-01312-s001.zip › molecules-2889197-supplementary.pdf]

SUPPORTING INFORMATION

Synthesis of α,ω-bis-Mercaptoacyl Poly(alkyl oxide)s and Development of Thioether Cross-Linked Liposome Scaffolds for Sustained Release of Drugs

Spyridon Mourtas [1,*], Georgios Kourmoulakis [2], Kremezis Stavros [2], Pavlos Klepetsanis [2,3] and Sophia G. Antimisiaris [2,3,*]

[1] Department of Chemistry, University of Patras, 26510 Rio Patras, Greece
[2] Laboratory of Pharmaceutical Technology, Department of Pharmacy, School of Health Sciences, University of Patras, 26510 Rio Patras, Greece; geokourmoulakis93@gmail.com (G.K.); stauros_chemeng@yahoo.com (S.K.); klepe@upatras.gr (P.K.)
[3] Institute of Chemical Engineering Sciences, Foundation for Research and Technology Hellas (FORTH/ICES), 26504 Rio Patras, Greece
* Correspondence: s.mourtas@upatras.gr (S.M.); santimis@upatras.gr (S.G.A.); Tel.: +30-2610-996-015 (S.M.); +30-2610-962-332 (S.G.A.)

1. NMR spectra, HPLC analysis of *S*-Mmt-mercapto acids

1.1 *S*-Mmt-2-mercaptopropionic acid (thiolactic acid) (6a)

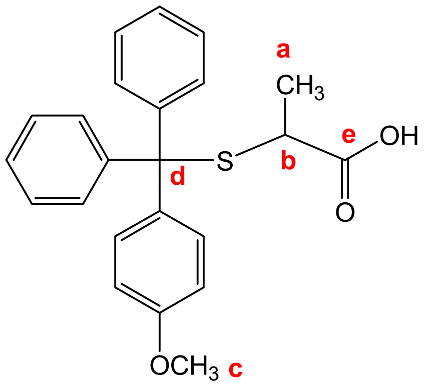

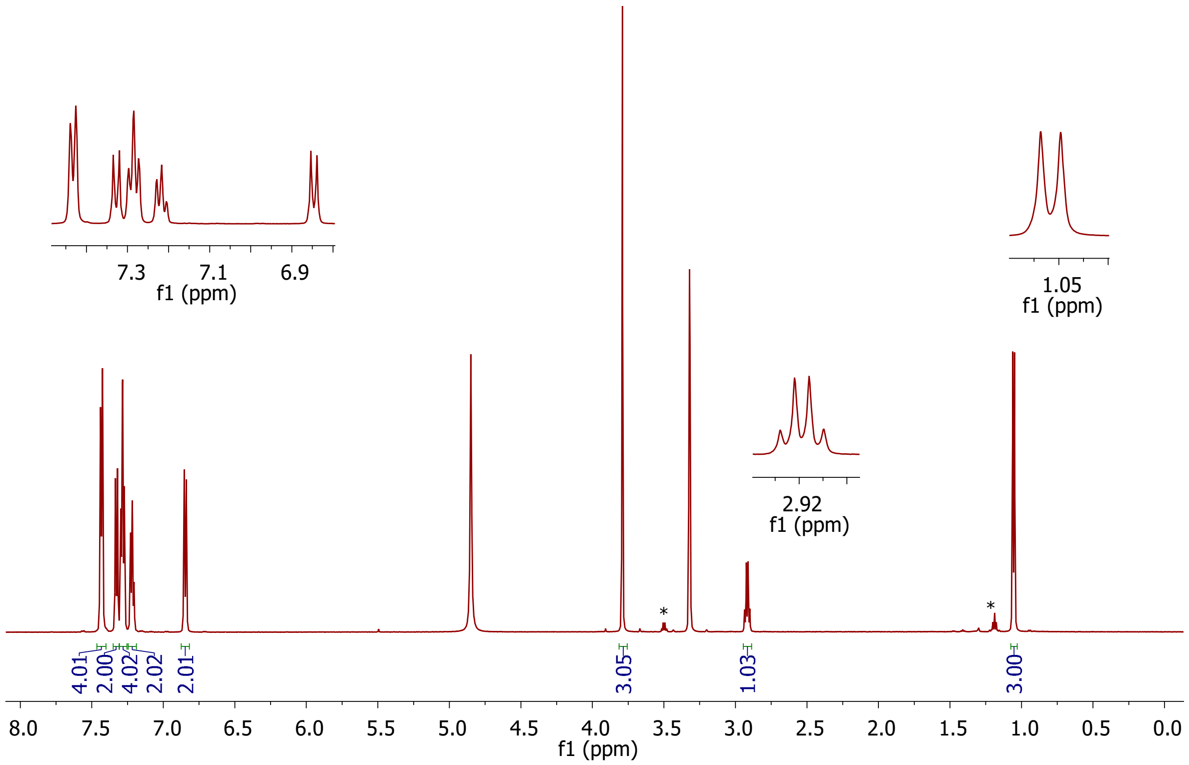

Figure S1. ^{1}H-NMR of *S*-Mmt-2-mercaptopropionic acid (thiolactic acid) (**6a**) in methanol-*d*4. *solvent residue

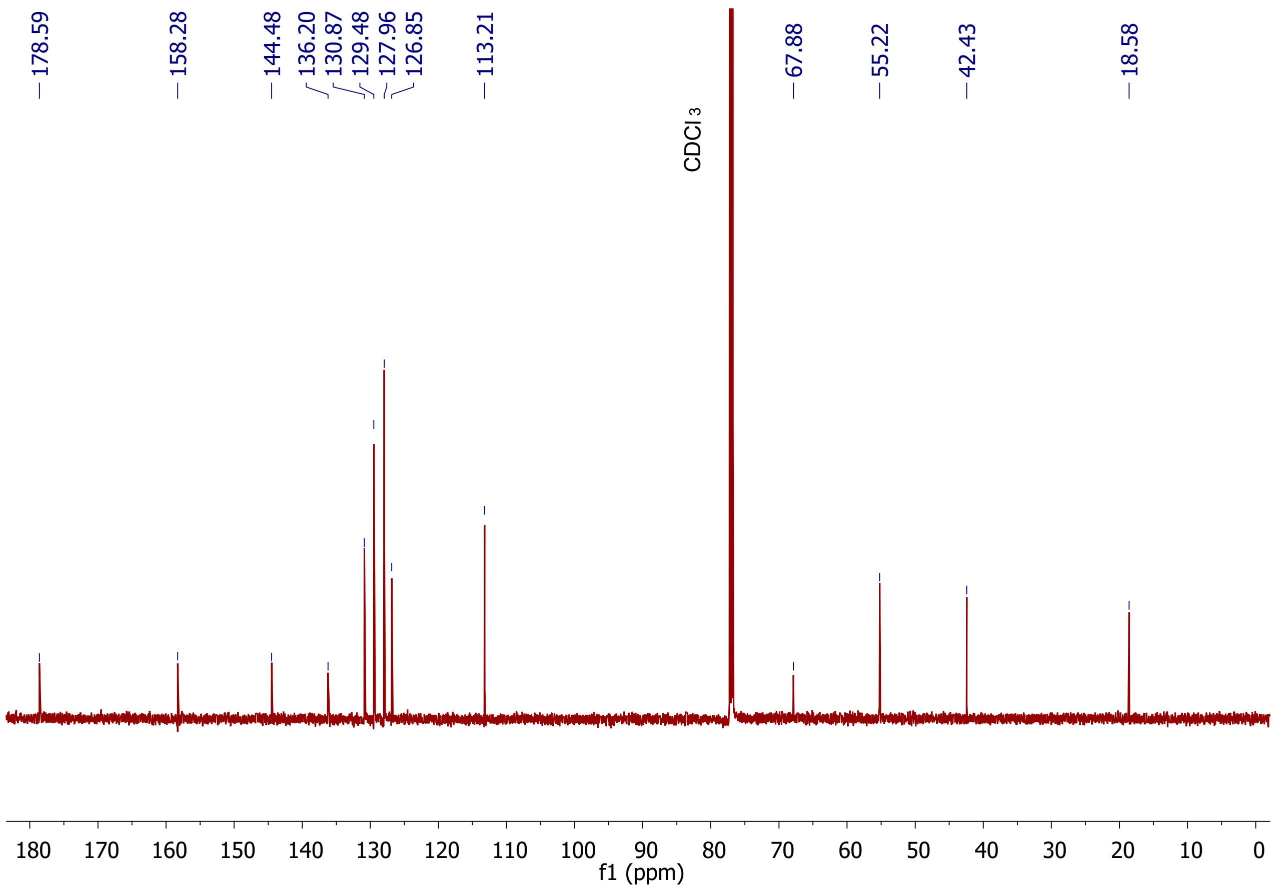

Figure S2. ^{13}C-NMR of *S*-Mmt-2-mercaptopropionic acid (thiolactic acid) (**6a**) in $CDCl_3$

1.2 *S*-Mmt-3-mercaptopropionic acid (6b)

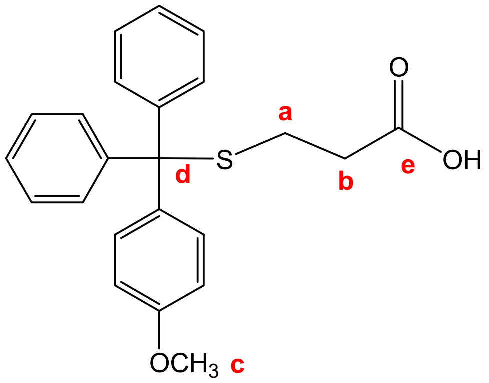

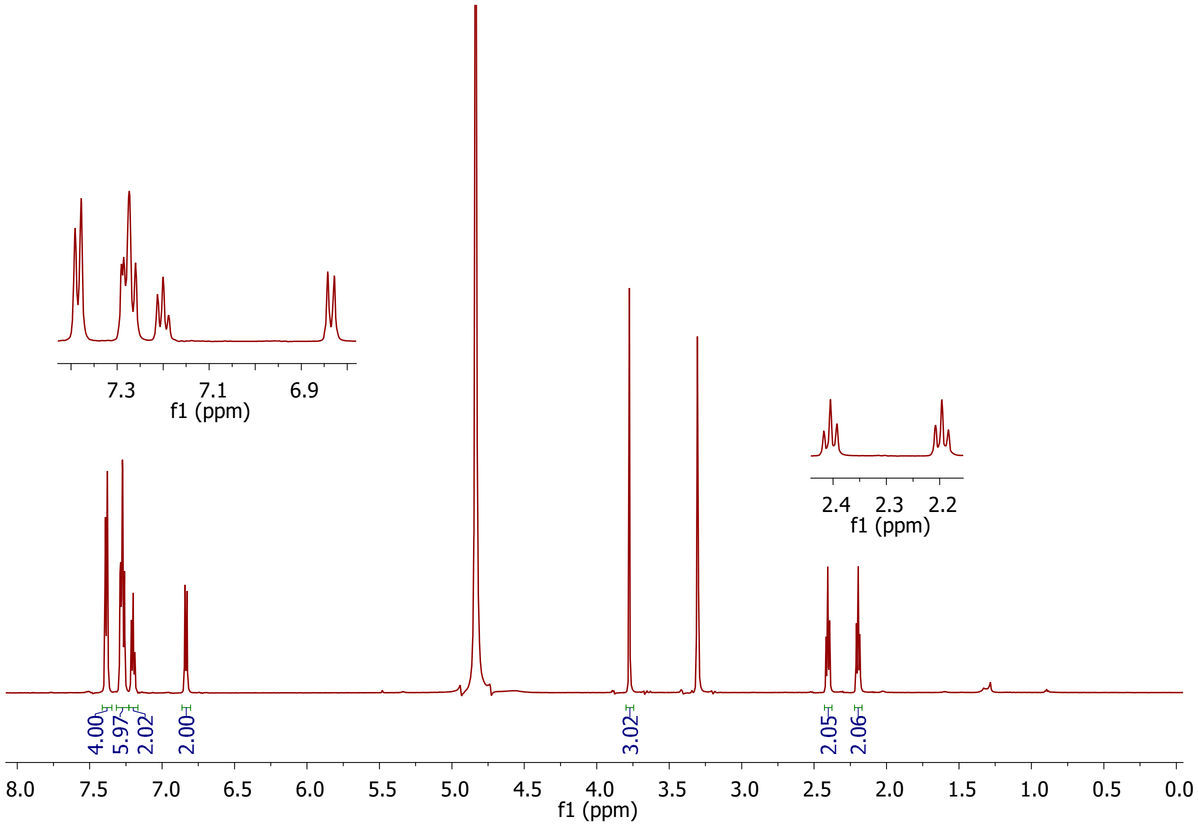

Figure S3. ^{1}H-NMR of *S*-Mmt-3-mercaptopropionic acid (**6b**) in methanol-*d*4.

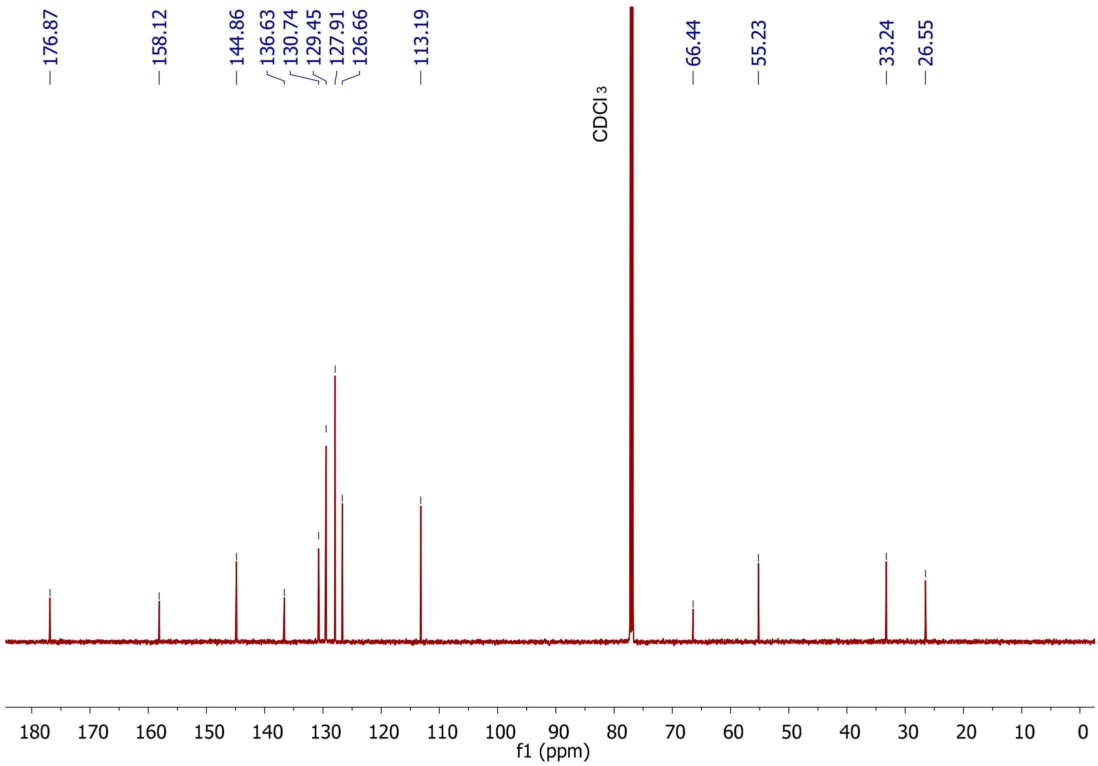

Figure S4. ^{13}C-NMR of *S*-Mmt-3-mercaptopropionic acid (**6b**) in $CDCl_3$.

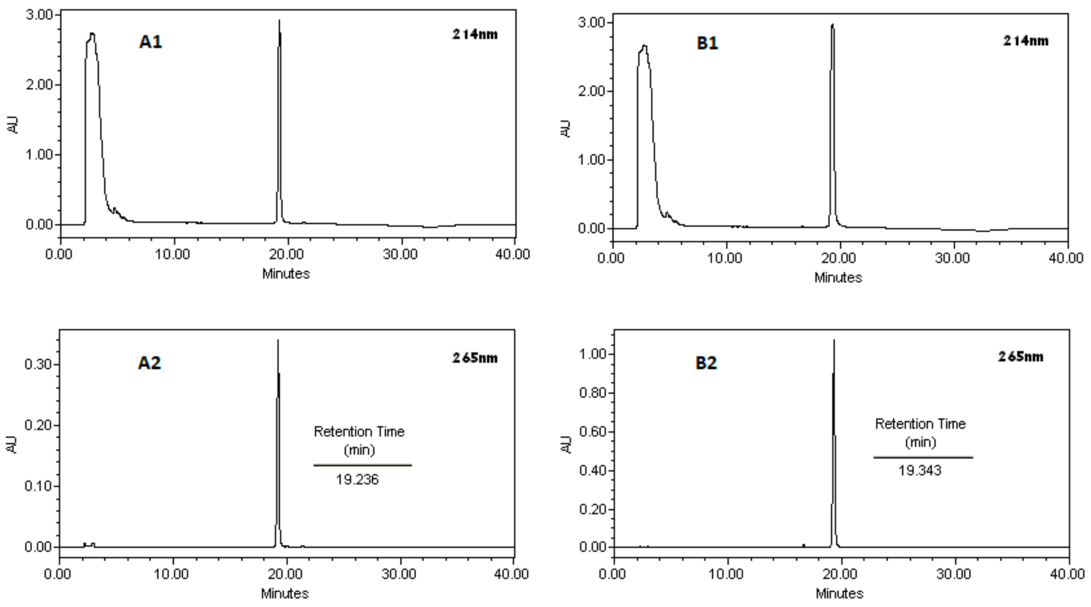

Figure S5. Analytical hplc of *S*-Mmt-2-mercaptopropionic acid (thiolactic acid) (**6a**) (A1, A2) and *S*-Mmt-3-mercaptopropionic acid (**6b**) (B1, B2) at 214 and 265 nm; column: LiChrospher 100, RP-18, 250-4, 5 µm; mobile phase solvents: AcCN/water (0.08% TFA); Gradient conditions: 20% to 100% AcCN in 30 min.

2. NMR spectra of α,ω-bis-mercaptoacyl poly(ethylene oxide)s

2.1 di-*S*-Mmt-di-2-mercaptopropionyl PEG10.000 (14)

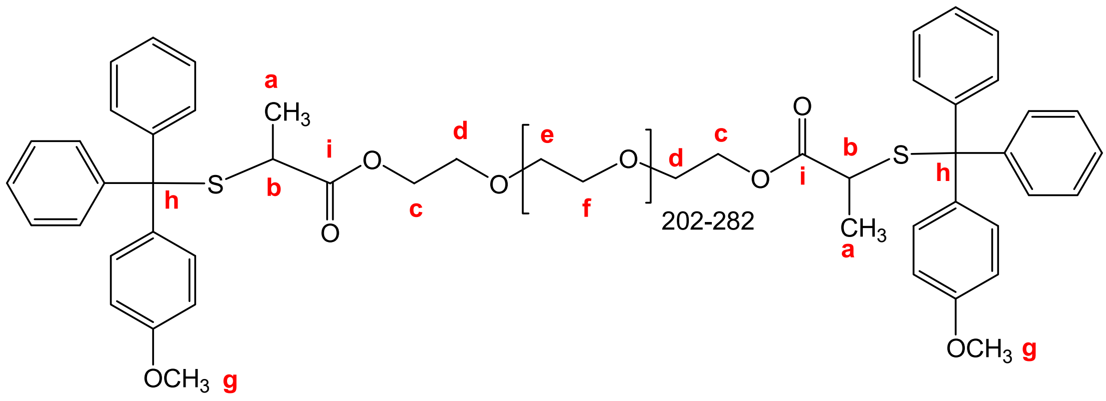

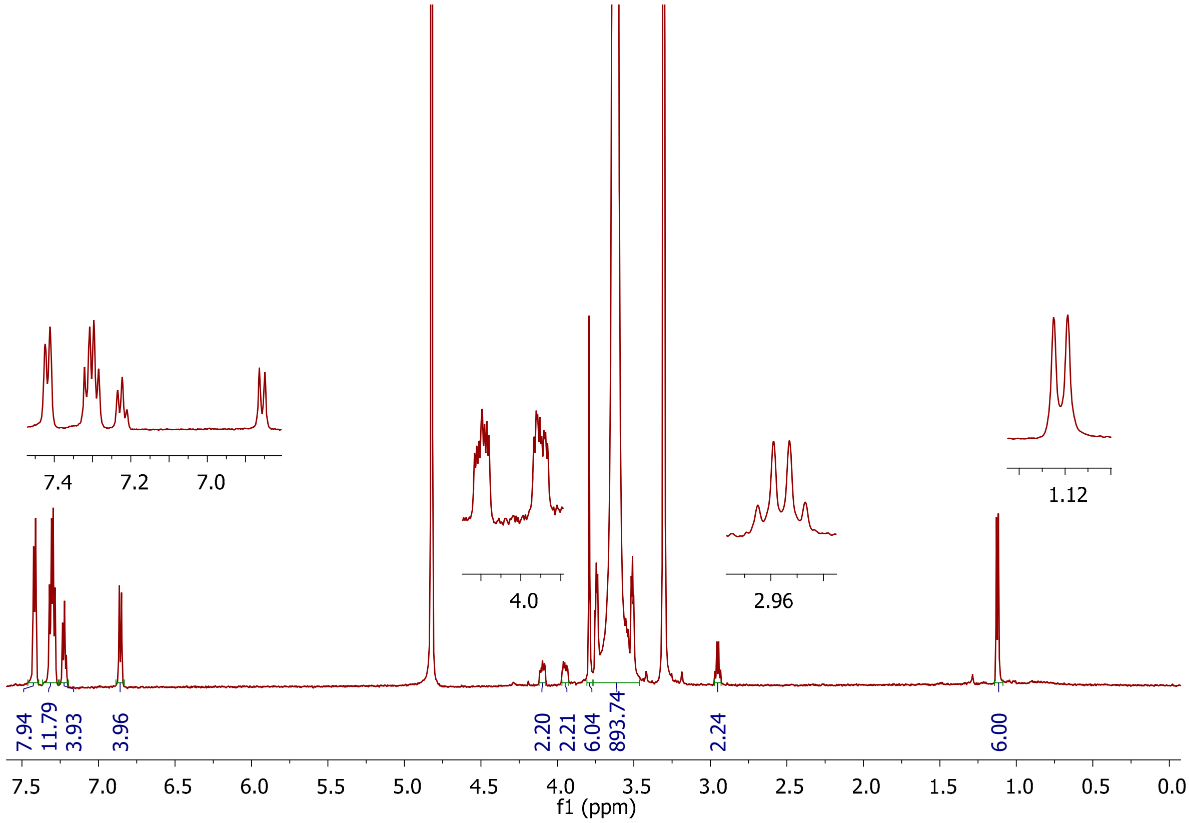

Figure S6. ^{1}H-NMR of di-*S*-Mmt-di-2-mercaptopropionyl PEG10.000 (**14**) in methanol-*d*4.

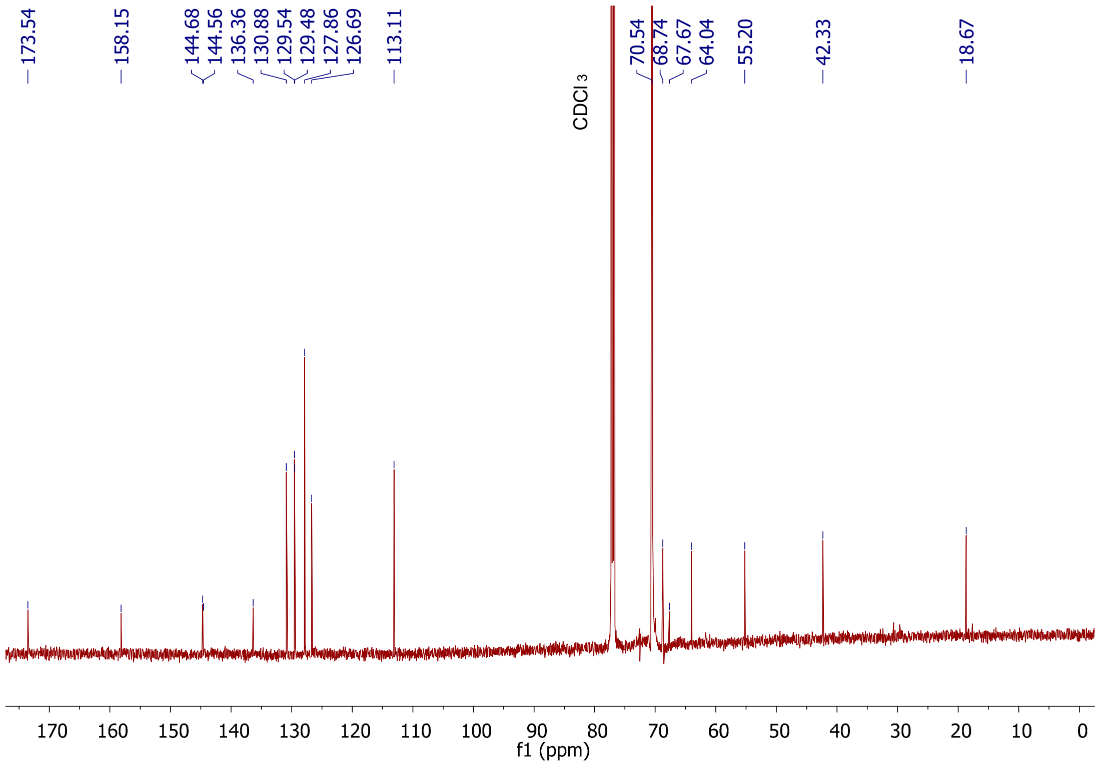

Figure S7. ^{13}C-NMR of di-*S*-Mmt-di-2-mercaptopropionyl PEG10.000 (**14**) in $CDCl_3$

2.2 di-2-mercaptopropionyl PEG10.000 (17)

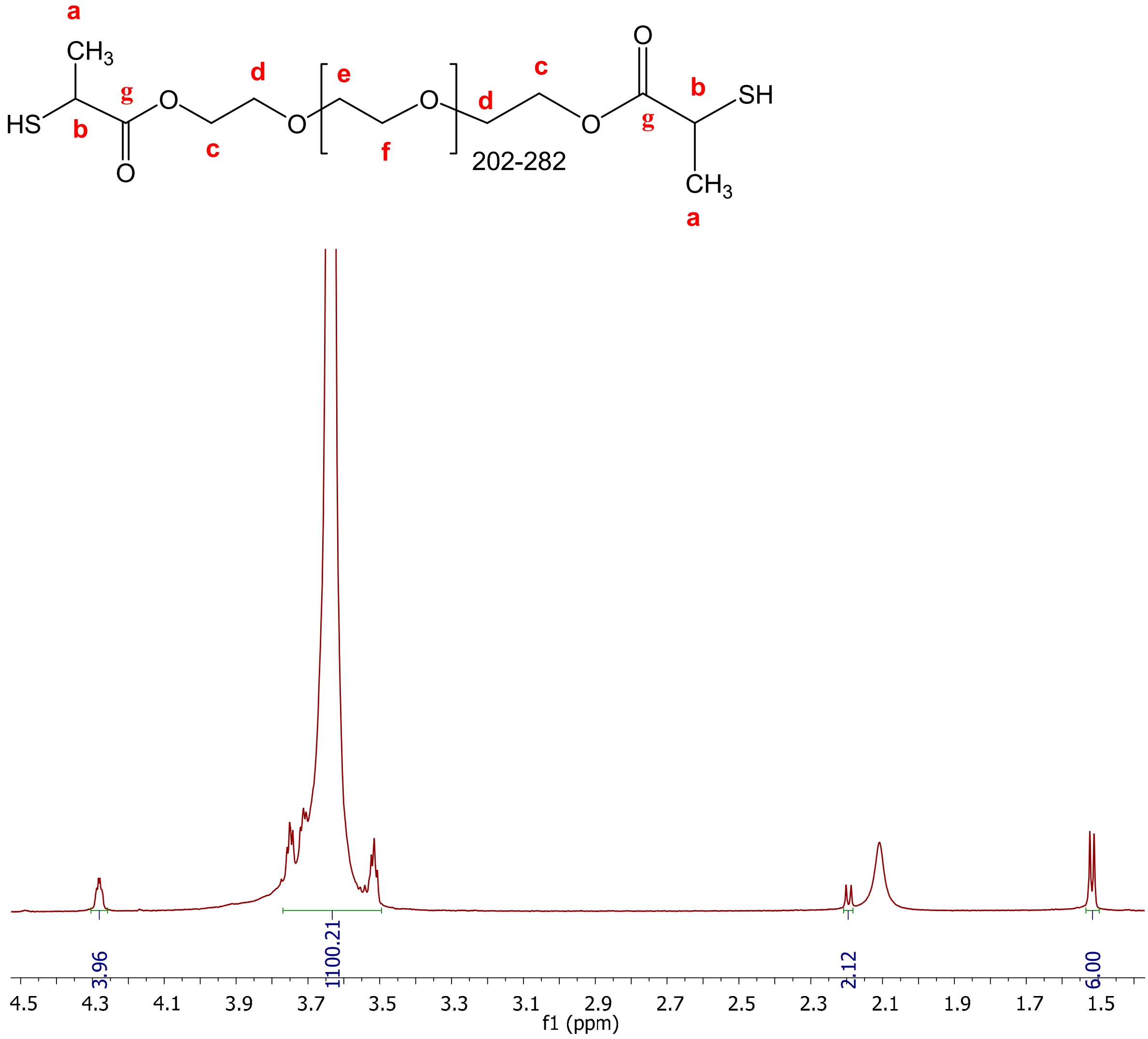

Figure S8. ^{1}H-NMR of di-2-mercaptopropionyl PEG10.000 (**17**) in $CDCl_3$.

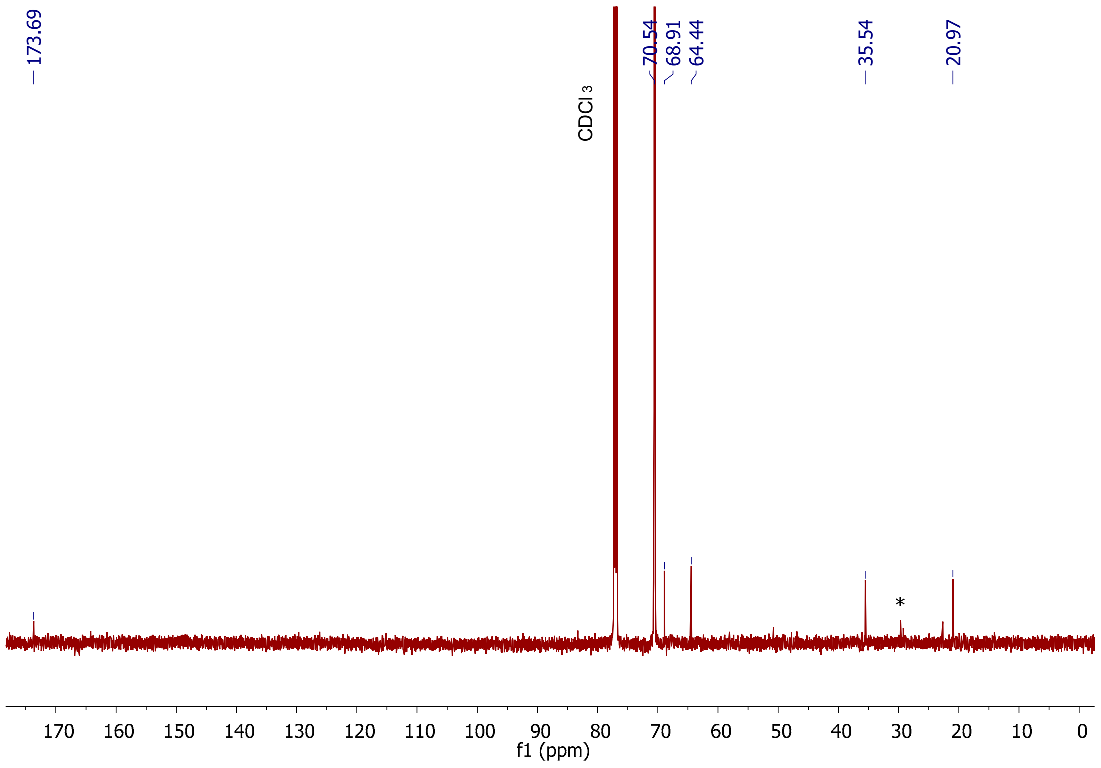

Figure S9. ^{13}C-NMR of di-2-mercaptopropionyl PEG10.000 (**17**) in $CDCl_3$; *solvent residue

2.3 di-*S*-Mmt-di-2-mercaptopropionyl PEG4.000 (15)

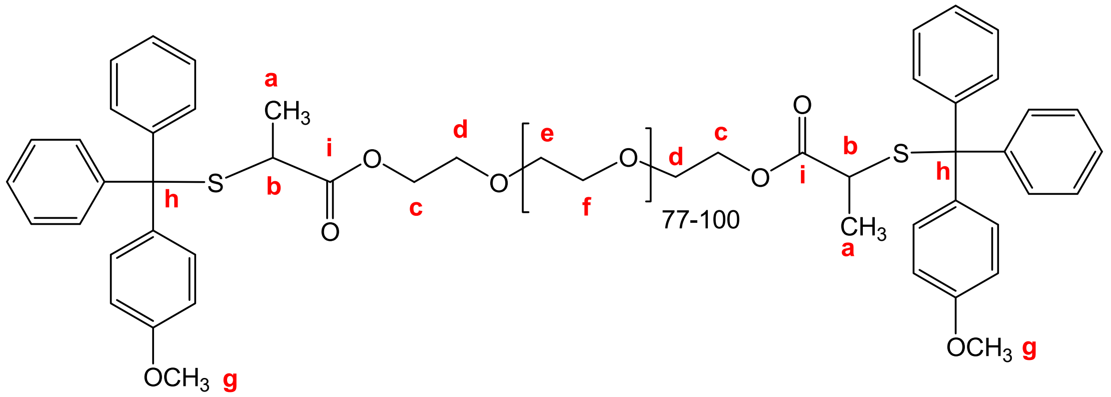

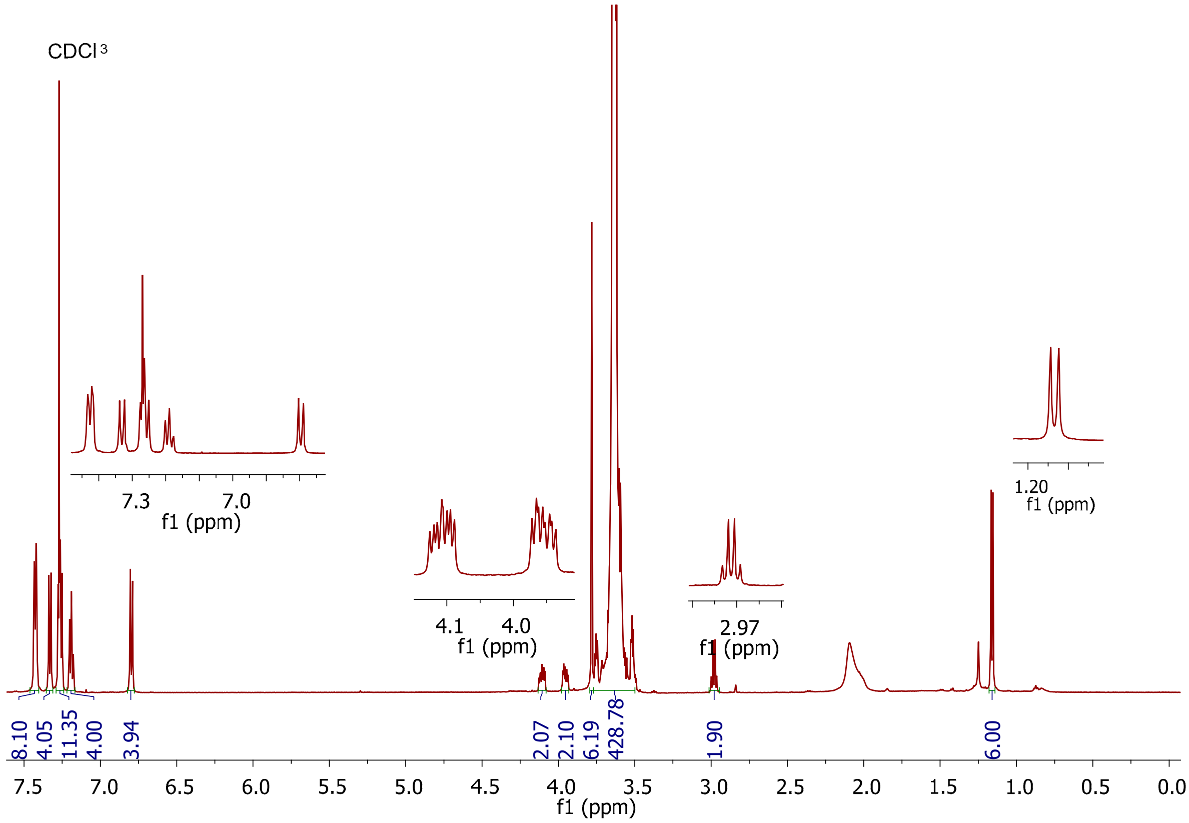

Figure S10. ^{1}H-NMR of di-*S*-Mmt-2-mercaptopropionyl PEG4.000 (**15**) in $CDCl_3$.

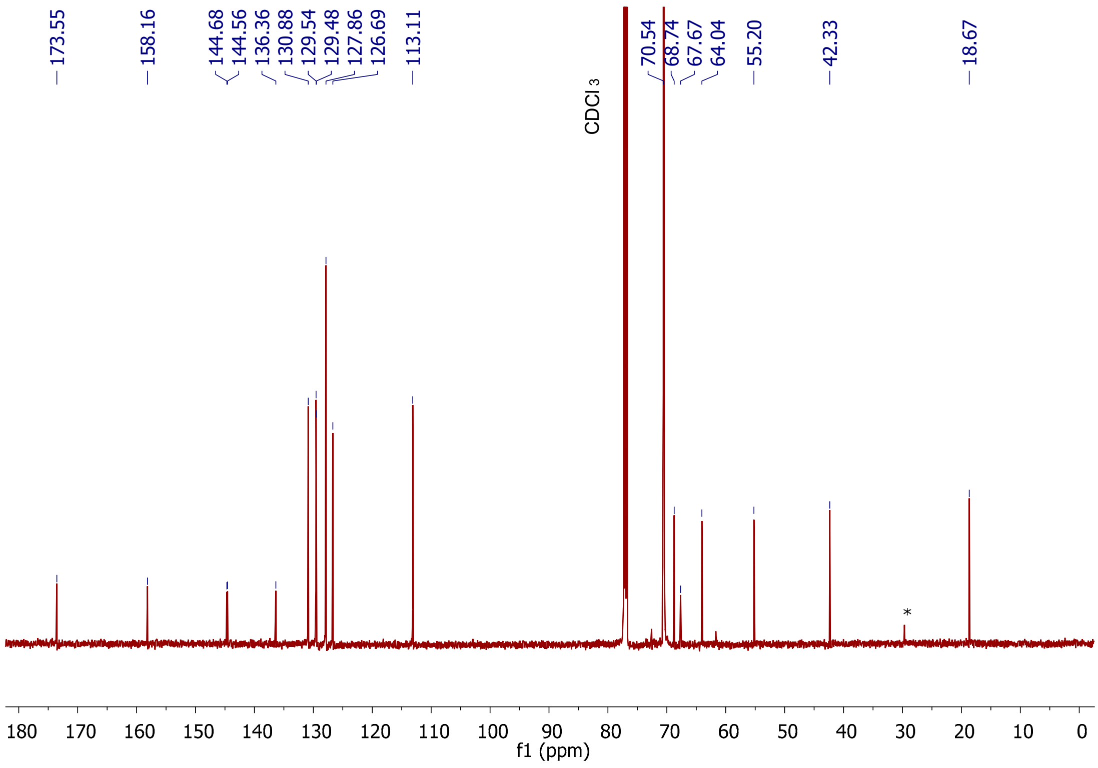

Figure S11. ^{13}C-NMR of di-*S*-Mmt-2-mercaptopropionyl PEG4.000 (**15**) in $CDCl_3$; *solvent residue

2.4 di-2-mercaptopropionyl PEG4.000 (18)

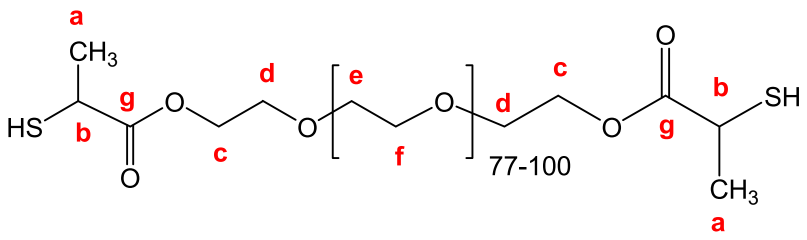

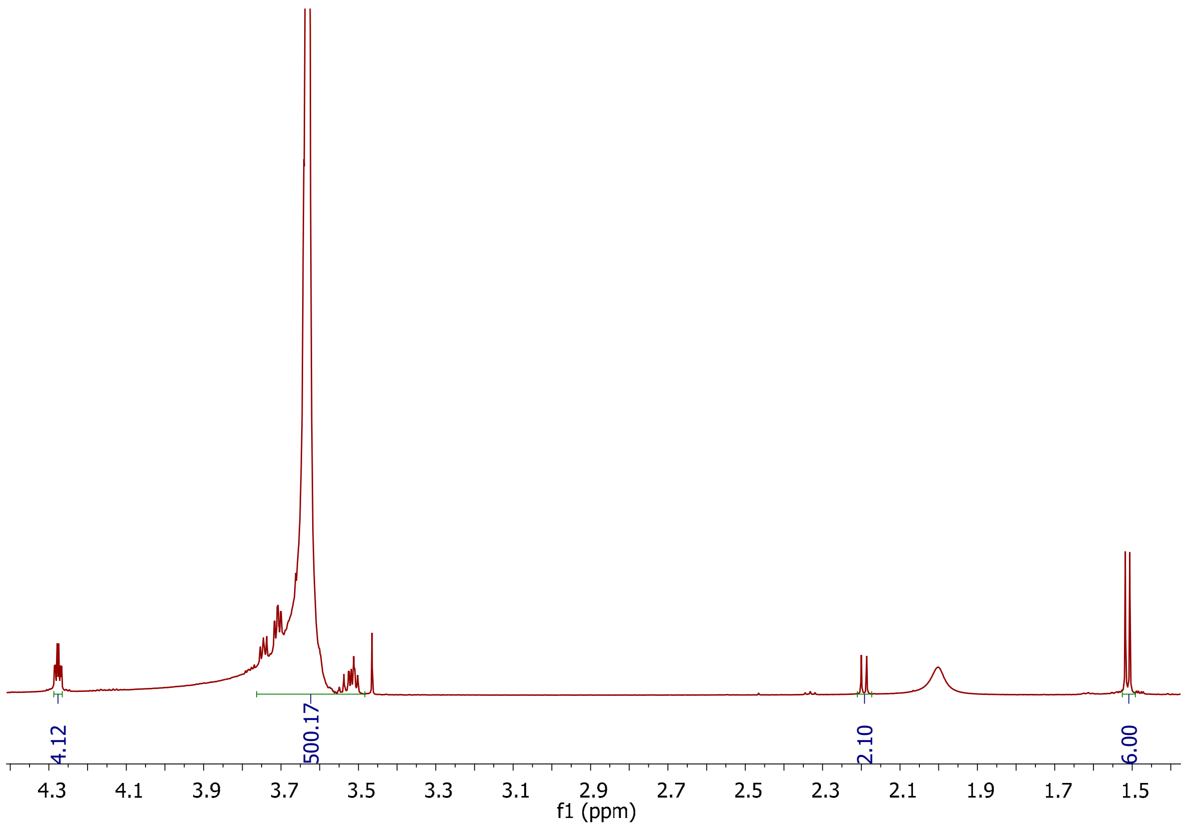

Figure S12. ^{1}H-NMR of di-2-mercaptopropionyl PEG4.000 (**18**) in $CDCl_3$.

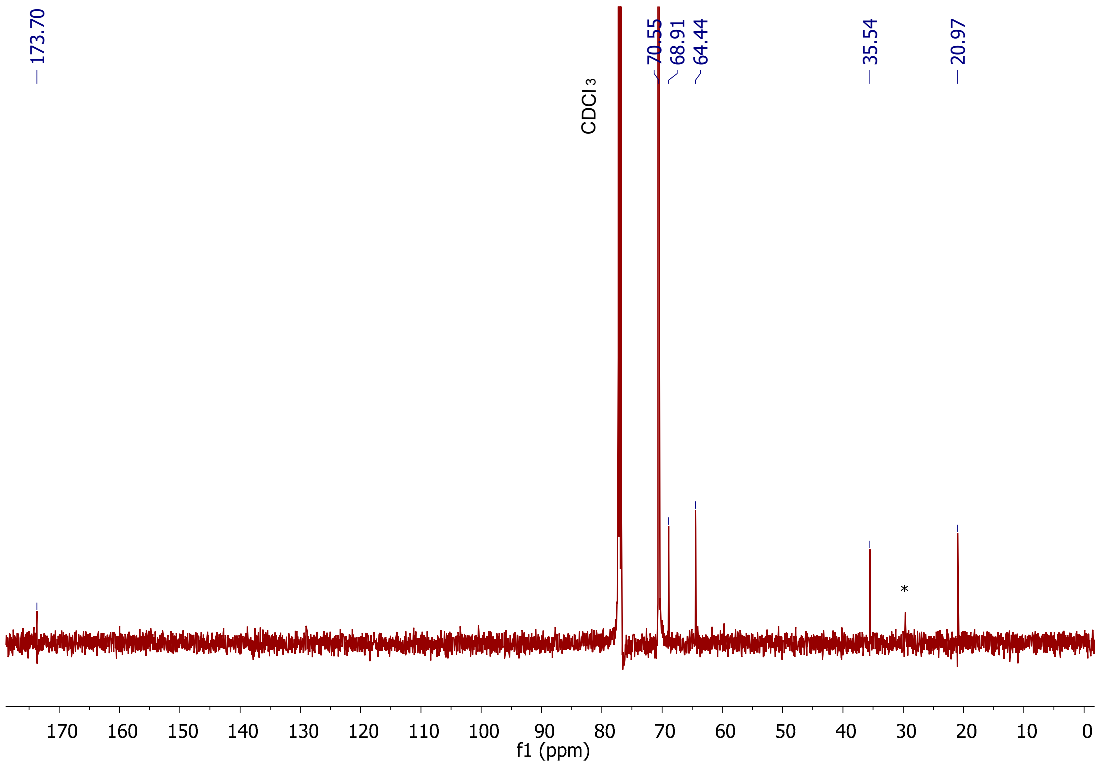

Figure S13. ^{1}H-NMR of di-2-mercaptopropionyl PEG4.000 (**18**) in $CDCl_3$; *solvent residue

2.5 di-2-mercaptopropionyl PEG1.000 (19)

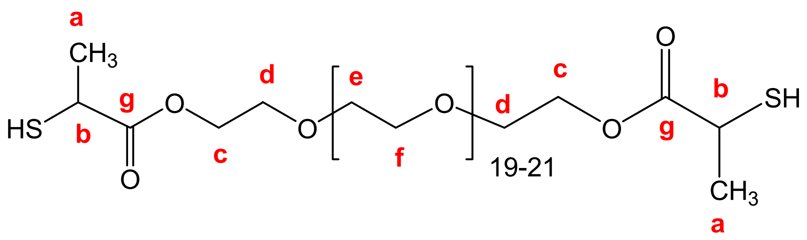

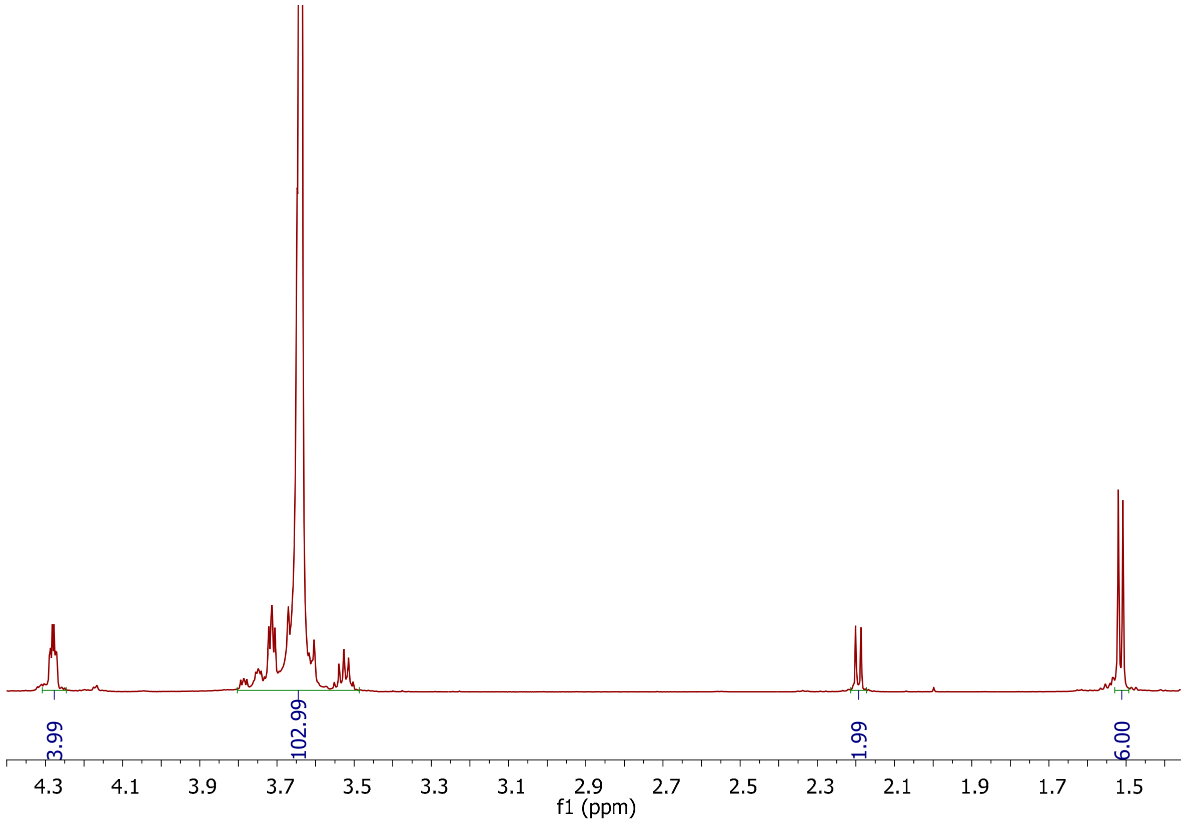

Figure S14. ^{1}H-NMR of di-2-mercaptopropionyl PEG1.000 (**19**) in $CDCl_3$.

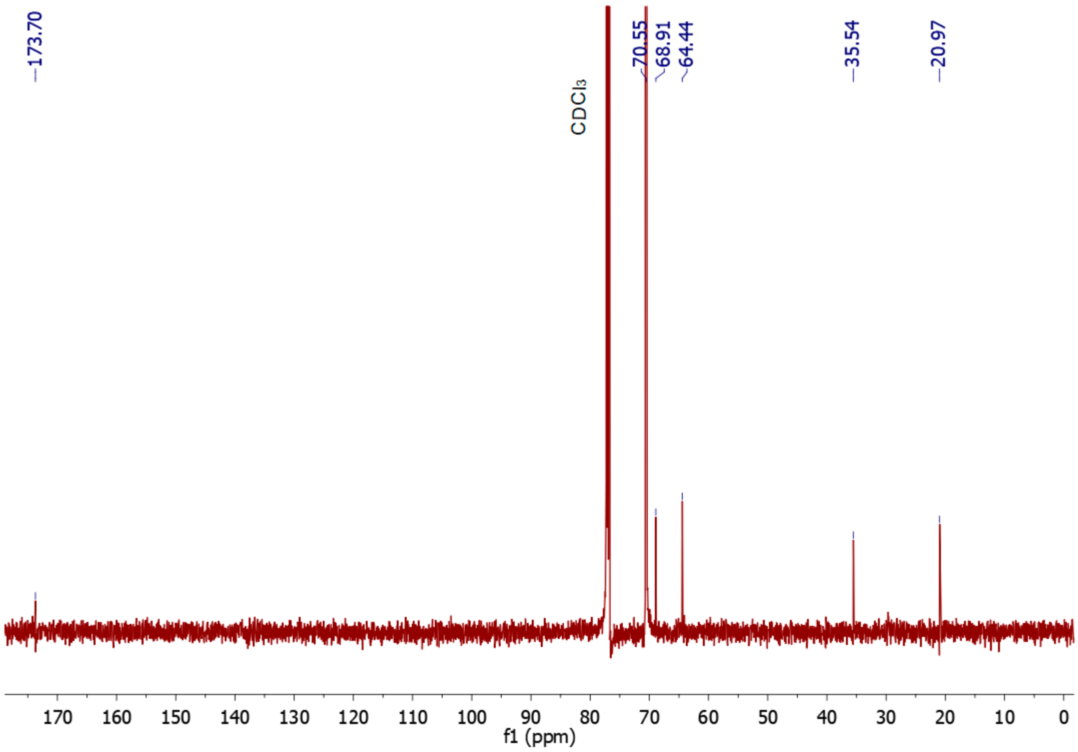

Figure S15. ^{1}H-NMR of di-2-mercaptopropionyl PEG1.000 (**19**) in $CDCl_3$.

3. ESI-MS spectra of α,ω-bis-mercaptoacyl poly(ethylene oxide)s

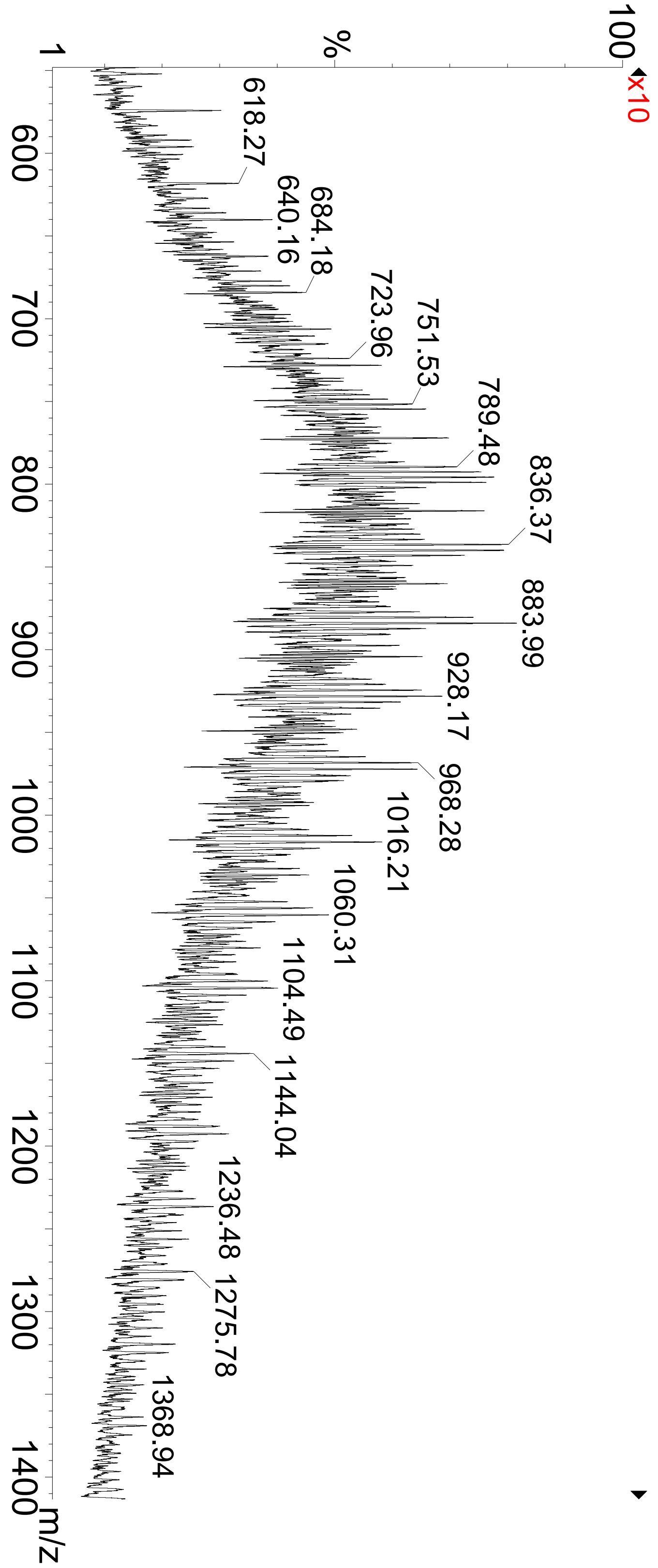

Figure S16. ESI-MS of di-2-mercaptopropionyl PEG10.000 (**17**); (M + 12H)/12.

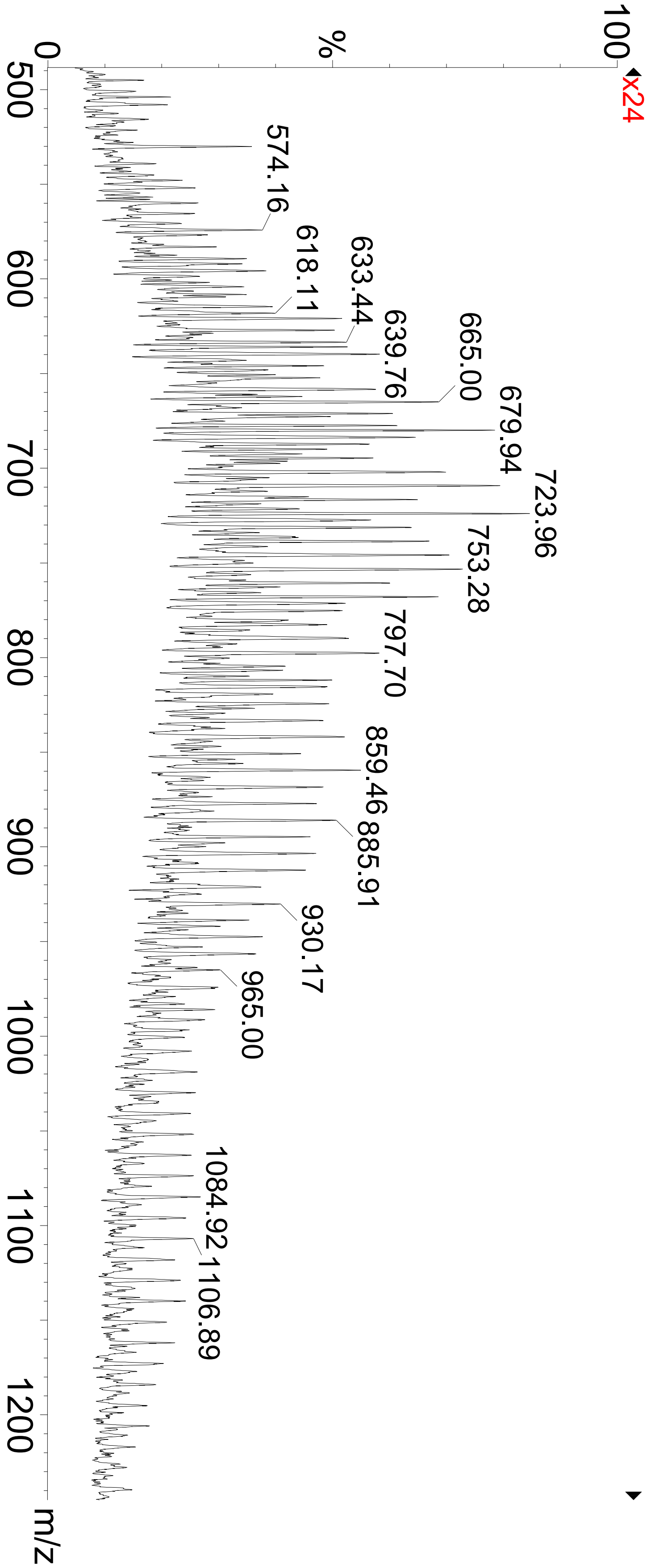

Figure S17. ESI-MS of di-2-mercaptopropionyl PEG4.000 (**18**); (M + 6H)/6.

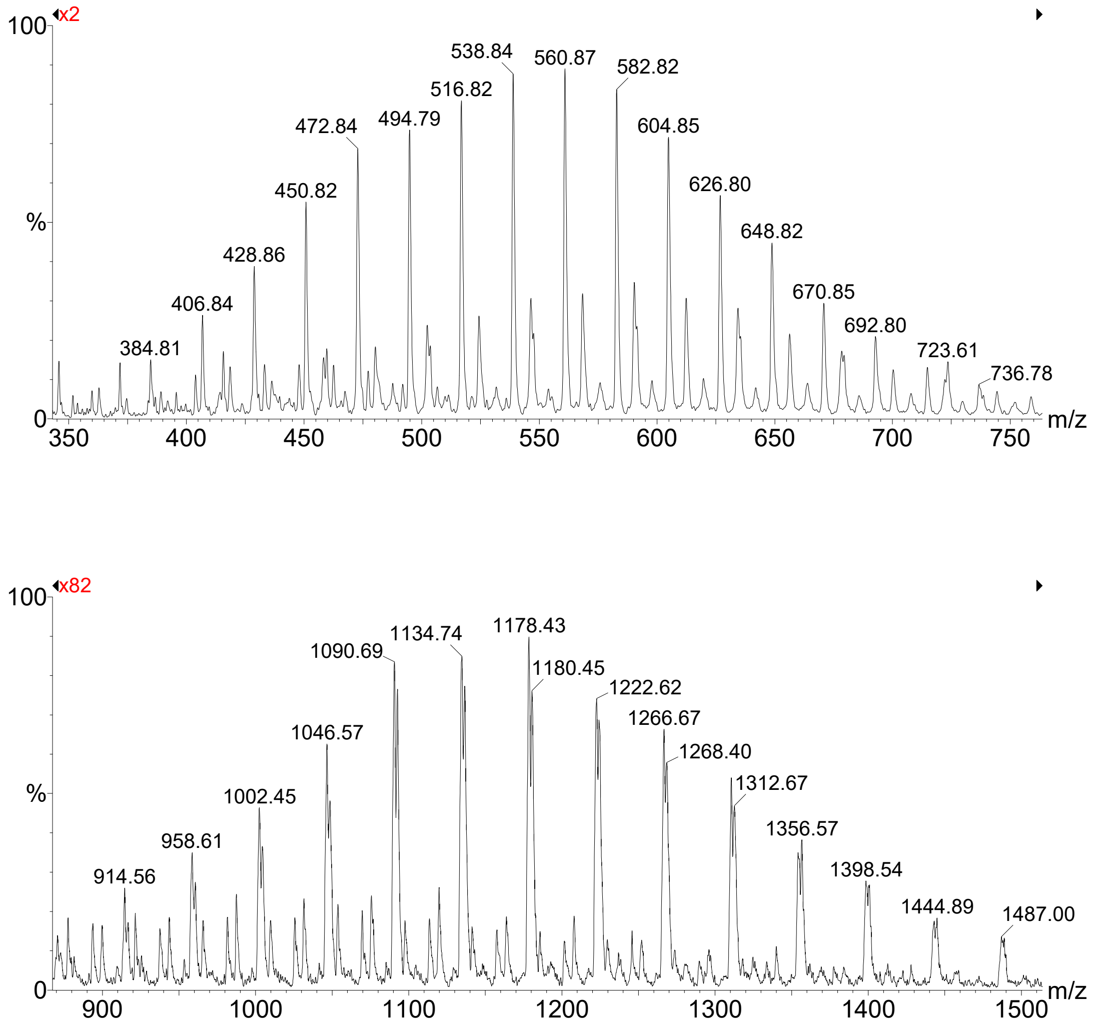

Figure S18. ESI-MS of di-2-mercaptopropionyl PEG1.000 (**19**); (M + 2H)/2, M + H.

4. NMR spectra of α,ω-bis-mercaptoacyl pluronic

4.1 di-*S*-Mmt-di-3-mercaptopropionyl pluronic (20)

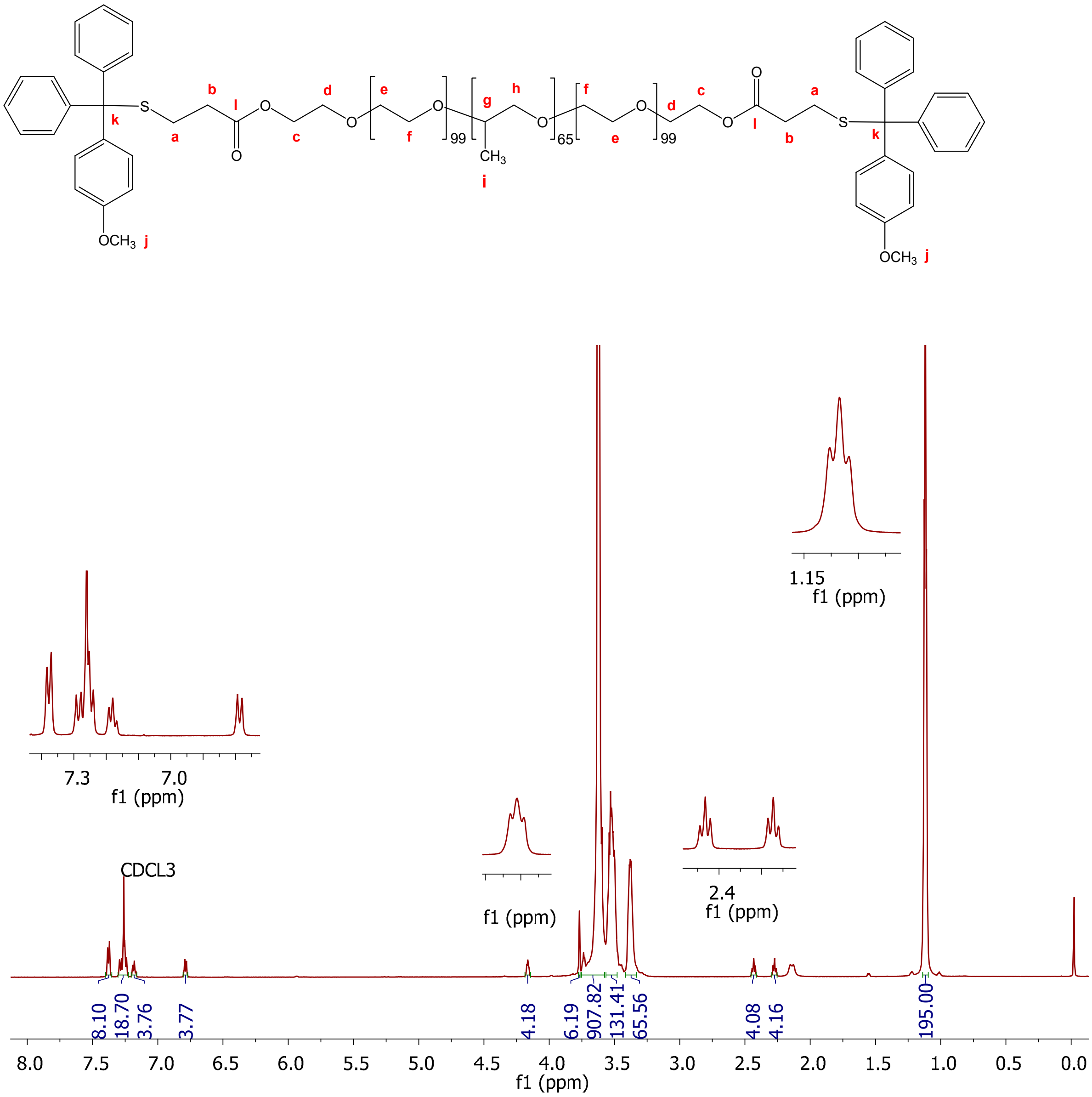

Figure S19. ^{1}H-NMR of di-*S*-Mmt-di-3-mercaptopropionyl pluronic (**20**) in $CDCl_3$.

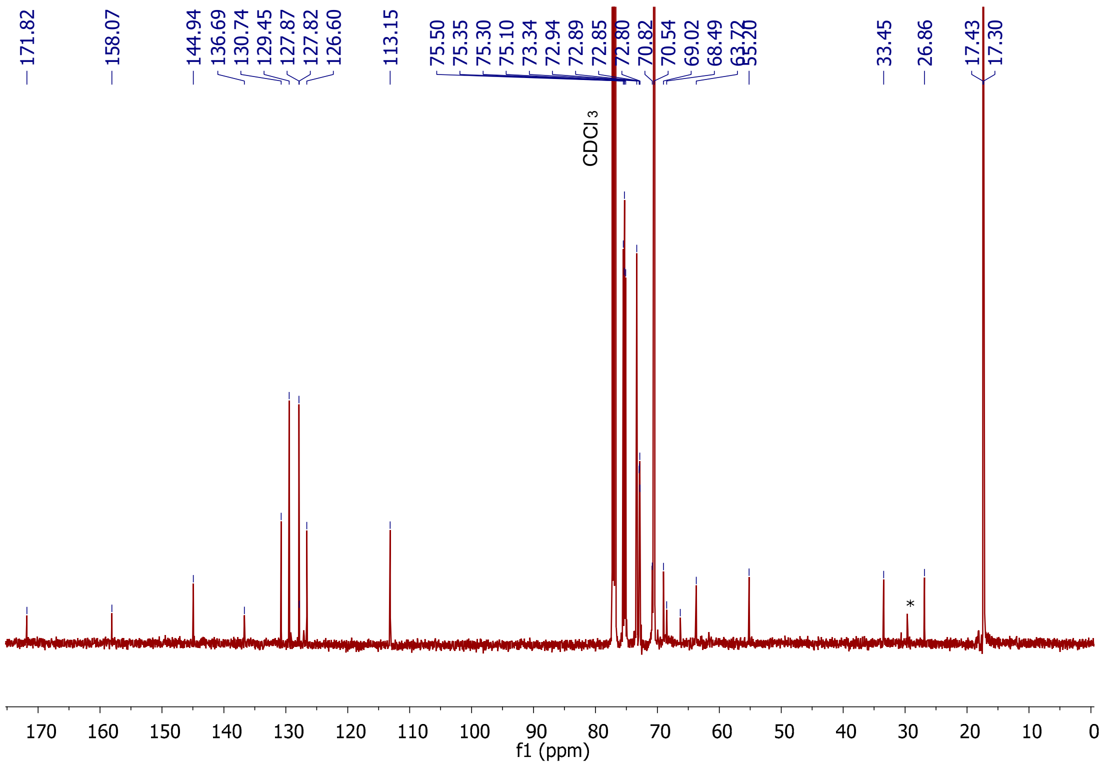

Figure S20. ^{13}C-NMR of di-*S*-Mmt-di-3-mercaptopropionyl pluronic (**20**) in $CDCl_3$; *solvent residue

4.2 di-3-mercaptopropionyl pluronic (21)

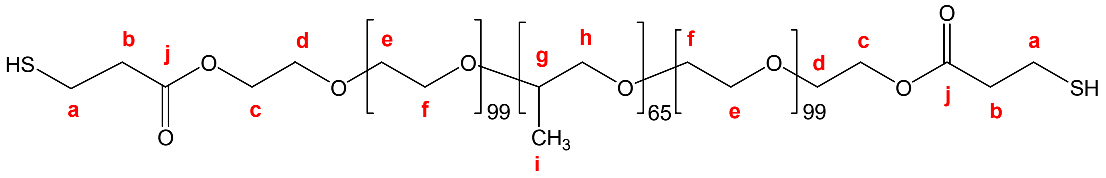

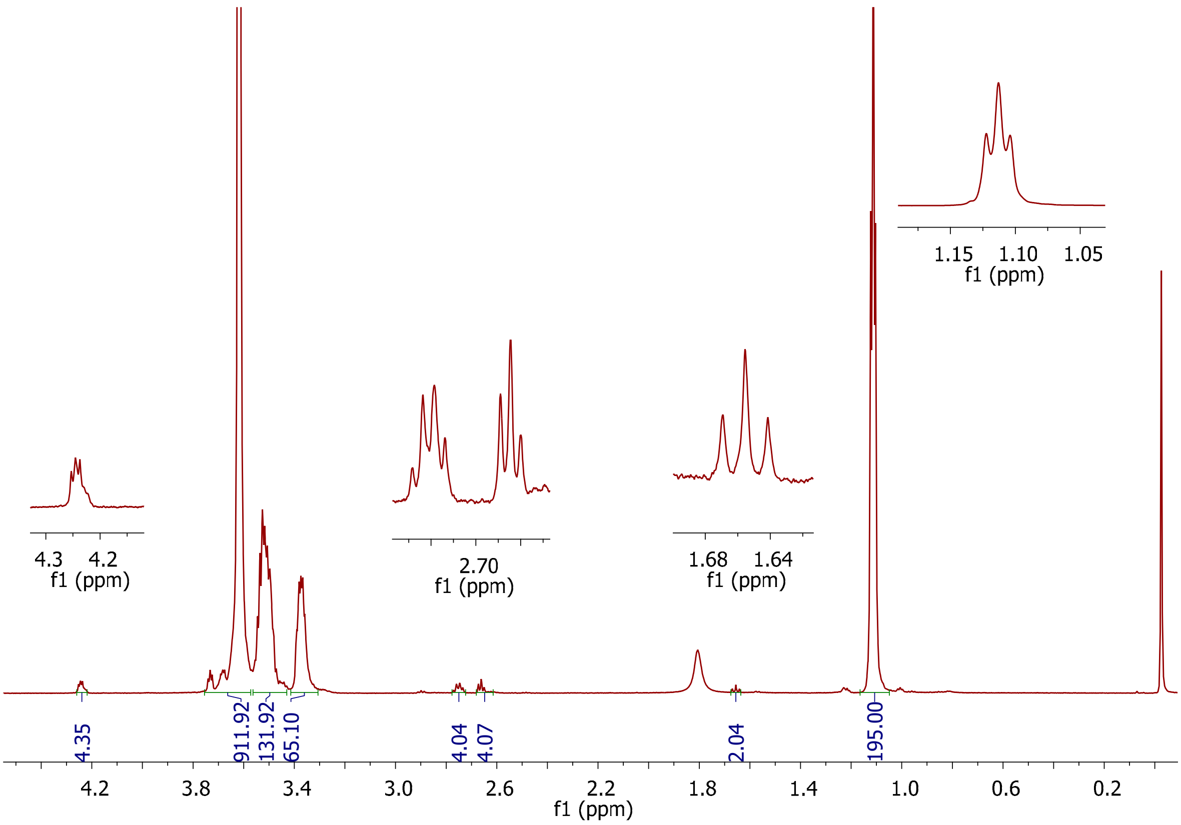

Figure S21. ^{1}H-NMR of di-3-mercaptopropionyl pluronic (**21**) in $CDCl_3$.

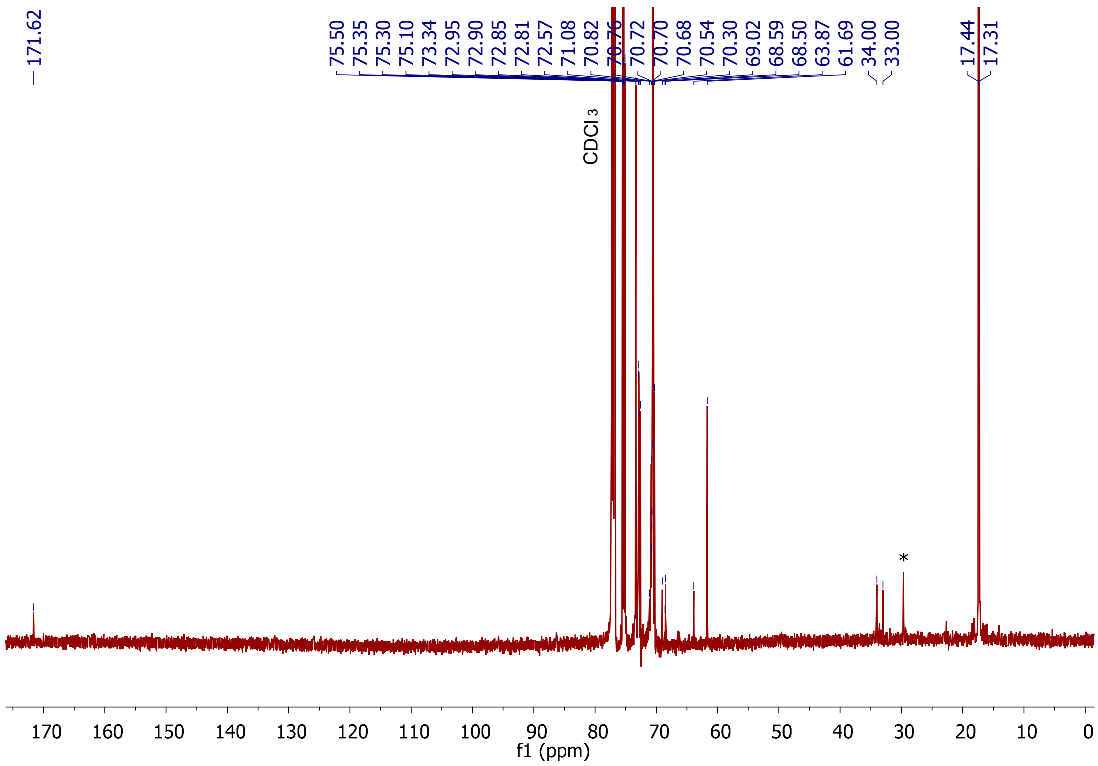

Figure S22. ^{13}C-NMR of di-3-mercaptopropionyl pluronic (**21**) in $CDCl_3$; *solvent residue

5. ESI-MS spectra of α,ω-bis-mercaptoacyl pluronic

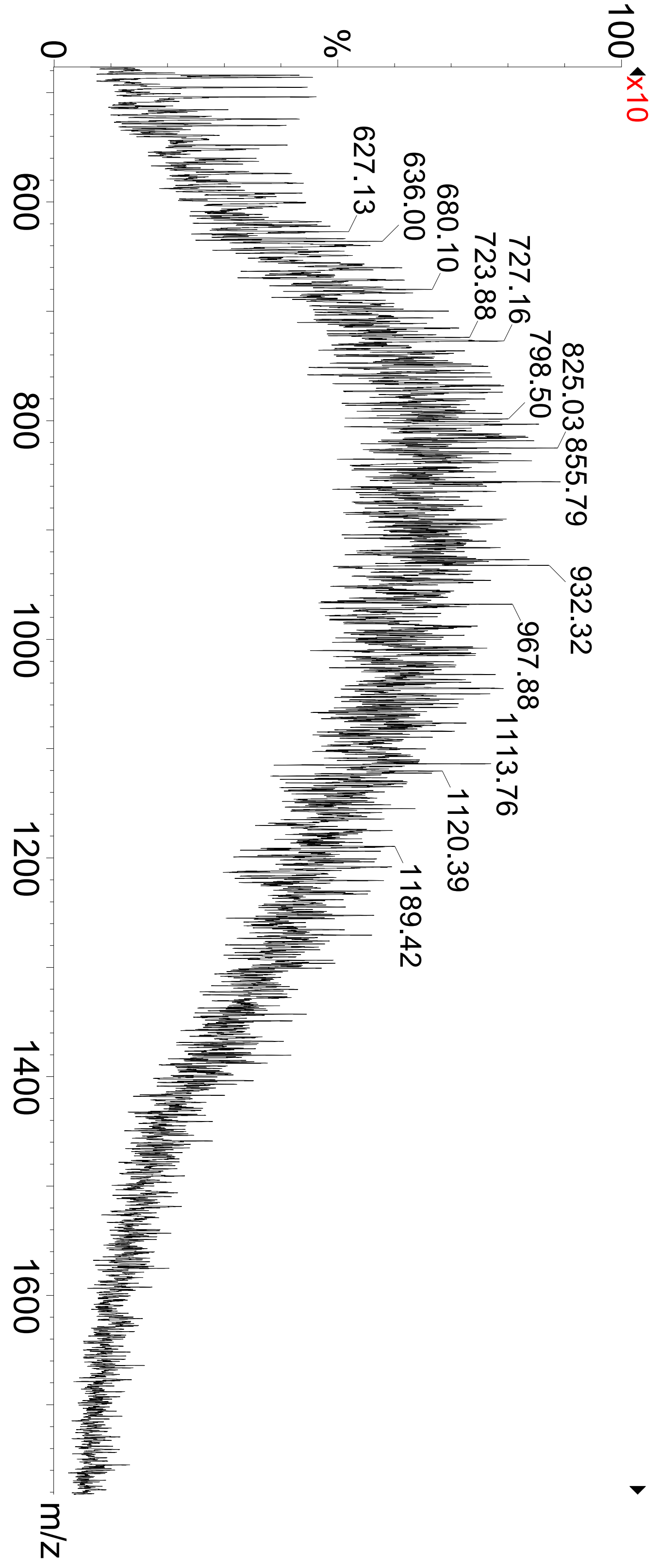

Figure S23. ESI-MS of di-3-mercaptopropionyl pluronic (**21**); M + 14H/14, M + 15H/15.

6. Real-time monitoring of the reaction progress between pre-formed PC-Lip-Mal and PEG-dithiol using 1D-CPMG ^{1}H-NMR

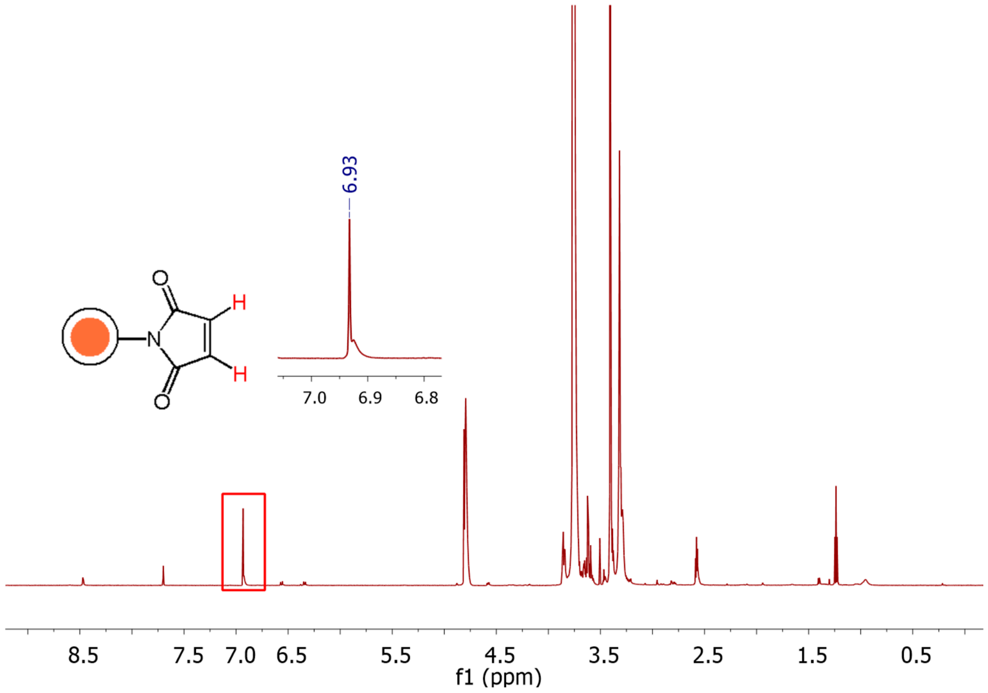

Figure S24. ^{1}H-NMR spectrum of PC/Chol/DSPE-PEG2000-Mal (2:1:0.03) mol/mol liposomes in D_2O using CPMG pulse sequence at 700 MHz, T = 27 °C. The lipid concentration was adjusted at 20 mg/mL (in D_2O).

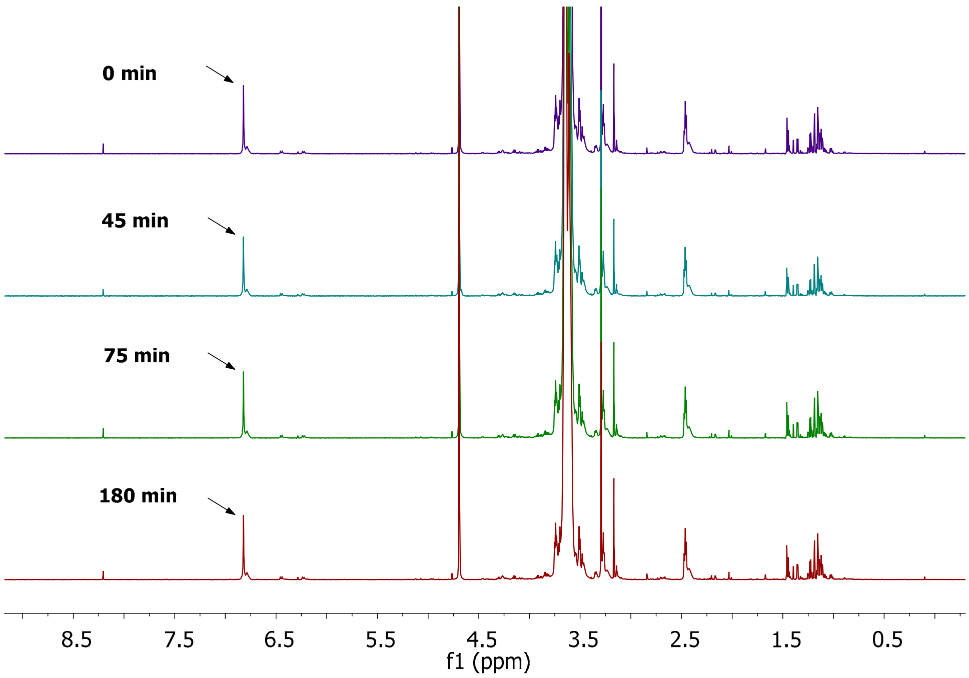

Figure S25. ^{1}H-NMR analysis of PC/Chol/DSPE-PEG2000-Mal (2:1:0.03) mol/mol liposomes in D_2O using CPMG pulse sequence at 700 MHz, T = 27 °C, at t = 0, 45, 75, 180 min. The lipid concentration was adjusted at 20 mg/mL (in D_2O).

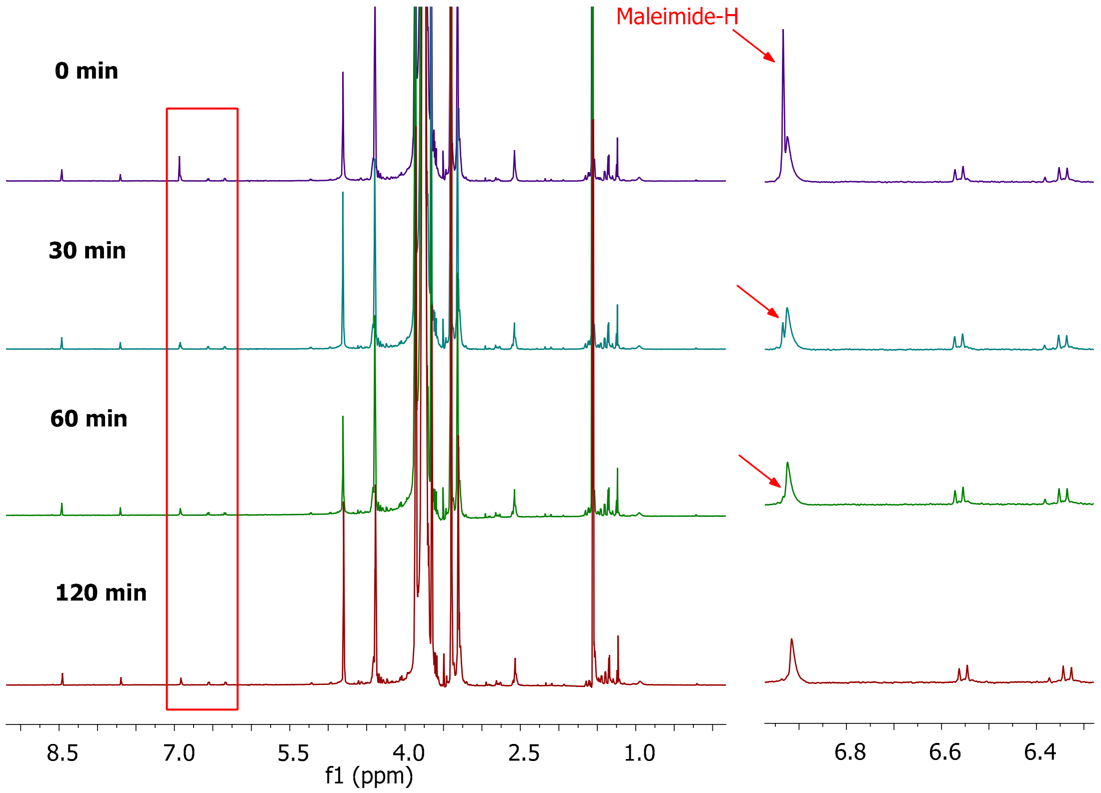

Figure S26. Real-time monitoring of the reaction progress between pre-formed PC-Lip-Mal and PEG-dithiol in D_2O using ^{1}H-NMR CPMG pulse sequence at 700 MHz, T = 27 °C. The lipid concentration was adjusted at 20 mg/mL (in D_2O).

7. Liposome physicochemical data before and after cross-linking reaction

7.1 Size distribution graphs for HPC-Lip-Mal before and after their reaction with PEG-dithiol.

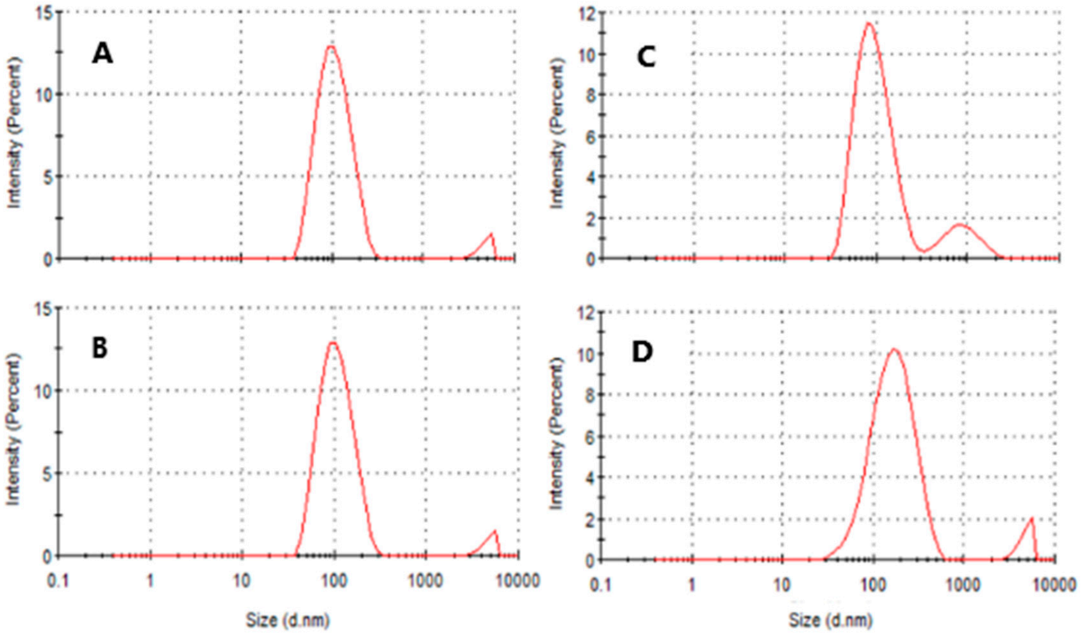

Figure S27. Size distribution before (**A**) and after reaction (**B**) for control liposomes. Size distribution before (**C**) and after reaction (**D**) for Lips-Mal.

8. Calcein Release from the liposomal structures in PBS

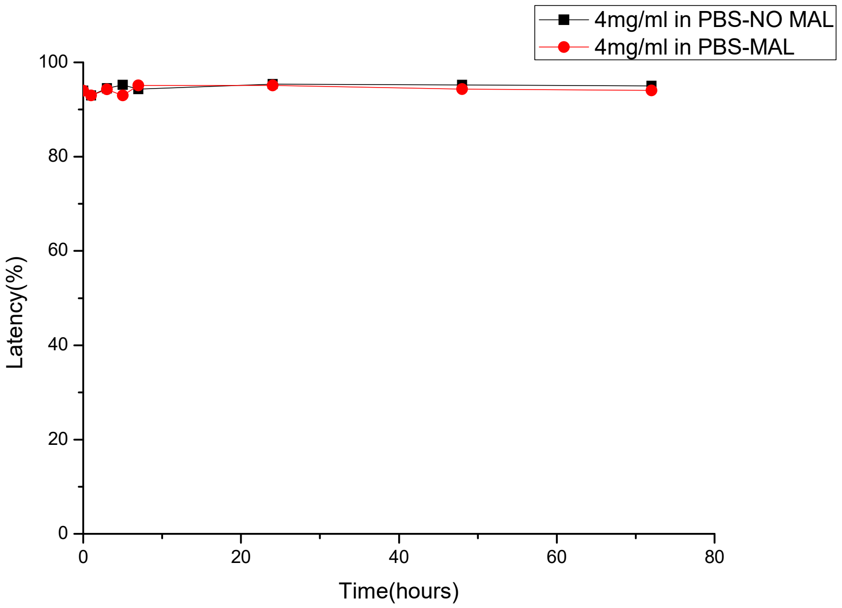

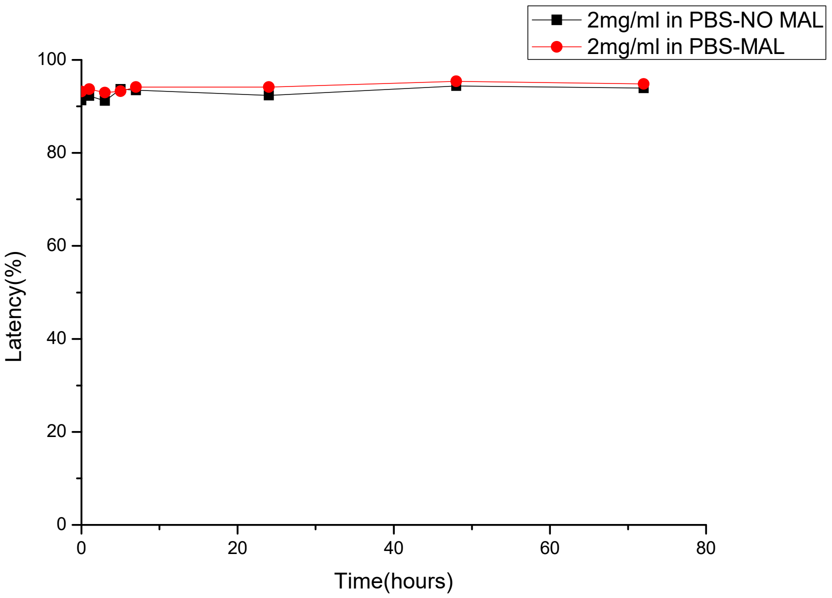

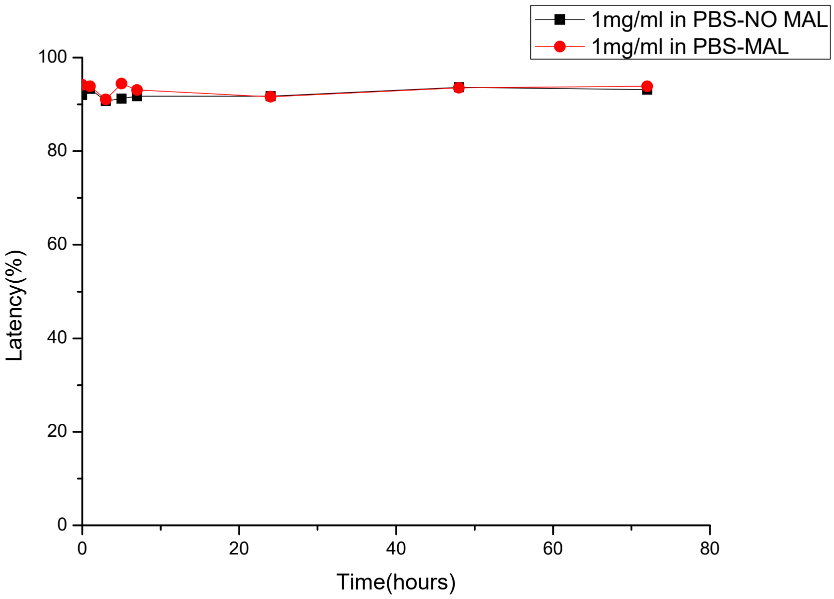
1mg/ml in PBS-NO MAL
1mg/ml in PBS-MAL
Latency(%)
Time(hours)
0
20
40
60
80
100

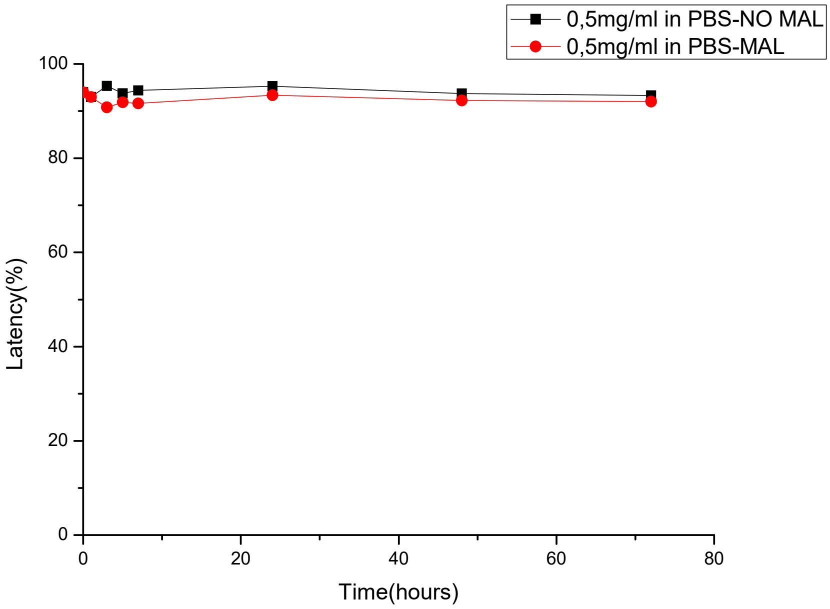
0,5mg/ml in PBS-NO MAL
0,5mg/ml in PBS-MAL
Latency(%)
Time(hours)
0
20
40
60
80
100

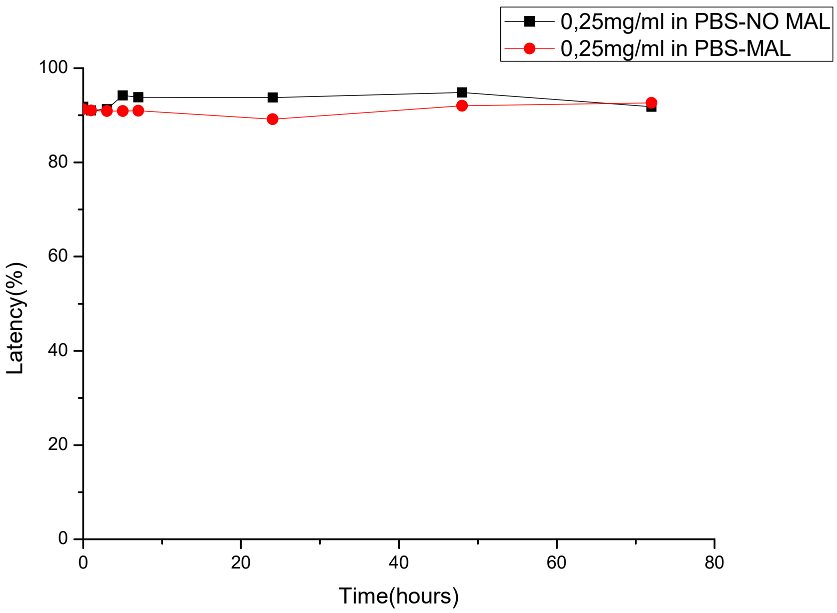

Figure S28. % Latency of calcein by the incubation of liposomes in PBS pH 7.40 at 37 °C. Control liposomes (HPC-Lip; PBS-NO MAL) and thioether cross-linked liposomes (HPC-LIP-di-thioether-PEG; PBS-MAL) at different concentrations (4.0 mg/mL, 2.0 mg/mL, 1.0 mg/mL, 0.5 mg/mL and 0.25 mg/mL).
